# Life events and change in support for political violence in the United States: findings from a 2023 nationally representative survey

**DOI:** 10.1186/s40621-025-00652-3

**Published:** 2025-12-27

**Authors:** Garen J. Wintemute, Sonia L. Robinson, Andrew Crawford, Elizabeth A. Tomsich, Mona A. Wright, Veronica A. Pear, Aaron B. Shev

**Affiliations:** 1 Department of Emergency Medicine, University of California Davis, 2315 Stockton Blvd, Sacramento, CA 95817 USA; 2 Centers for Violence Prevention, University of California Davis, Sacramento, CA USA

**Keywords:** Political violence, Firearm violence, Domestic violent extremism, Terrorism, Life events

## Abstract

**Background:**

A nationally representative longitudinal survey in the USA found a decrease in population-level support for political violence from 2022 to 2023. This individual-level analysis of those data examines associations between the occurrence of 18 specified life events and subsequent change in views on political violence.

**Methods:**

Participants in the Life in America Survey were members of the Ipsos KnowledgePanel. Wave 2 of the survey was fielded online May 18-June 8, 2023; all respondents to 2022’s Wave 1 who remained in KnowledgePanel were invited to participate. We calculated individual scores for 2022 and 2023 on 35 political violence measures from the first component of an ordinal principal components analysis and computed the difference in scores for individual respondents from 2022 to 2023 to represent a 1-year change in these measures. Our principal outcomes are adjusted mean differences in change scores from 2022 to 2023 between individuals experiencing and not experiencing the 18 life events.

**Results:**

The completion rate was 84.2%; there were 9385 respondents. Support for political violence decreased for 19.9% of respondents, increased for 14.2%, and remained unchanged for 65.9%. When events were considered individually in a model that adjusted for sociodemographic characteristics and other life events, only “things improved for me financially” was associated with decreased support for political violence among respondents as a whole; “I gave up on politics” was associated with an increase. No event was associated with change among both men and women when they were analyzed separately. Among respondents who reported in 2022 that violence was usually or always justified for at least 1 political objective, no events were associated with change in support for political violence. Among those who strongly approved in 2022 of left-wing violent extremist organizations or movements, “my political beliefs changed a lot” was associated with a large decrease.

**Conclusions:**

In this cohort, few life events were associated with changes in support for political violence across the entire population, but there were important subset findings. The findings support interventions to improve measures of economic well-being across the population and to encourage belief change among extremists as political violence prevention measures.

**Supplementary Information:**

The online version contains supplementary material available at 10.1186/s40621-025-00652-3.

## Background

Violence is a health problem, and for several years there has been widespread concern among researchers, policy experts, and law enforcement professionals that the United States is at risk for political violence in the near future [1–8]. That concern has increased in 2025, driven by new instances of political violence and the current federal administration’s reaction to them, which includes deployment of the National Guard and regular armed forces against civilians [9–11].

In 2022, we initiated the Life in America Survey, a large, annual, nationally representative longitudinal exploration of Americans’ views on democracy and support for political violence [12, 13]. The survey addresses support for political violence in general and to advance specified political objectives (such as “to stop people who do not share my beliefs from voting”); personal willingness to engage in political violence at varying levels of severity (from property damage to homicide) and against specified target populations (such as elected officials); and expectations of firearm use in committing political violence.

The 2022 survey (Wave 1) yielded concerning findings, among them that 32.8% of respondents considered violence usually or always justified to advance at least 1 of the specified political objectives [12]. Encouragingly, 2023’s Wave 2 indicated improvement on this measure, with 25.3% of respondents considering violence usually or always justified to advance any of the specified objectives [13]. Such population-level findings provide little basis for understanding why individuals become less or more supportive of political violence, however.

The literature on individuals no longer involved in extremist or terrorist violence has associated specific life changes and events with that disinvolvement [14–19]. Among them are disillusionment, disaffection from an ideology or belief system, family obligations, new or better employment, and other new social or economic opportunities. More broadly, life course research has reinforced the importance of adult life events in altering individual trajectories of criminal behavior [20–22].

We designed Wave 2 in part to address the question of whether experiencing specified life events was associated with change in support for political violence at the individual level. Wave 2 was conducted in May-June 2023. It repeated Wave 1’s questions on support for and willingness to engage in political violence and collected data on whether respondents experienced any of 18 major life events—primarily events identified in the literature on disinvolvement from extremist or terrorist violence—in the year following their participation in Wave 1. Because this longitudinal survey provides linked individual responses, we are able to compare individual change scores on our political violence outcome measures between individuals who experience and do not experience the specified life events (our exposures of interest) during the time between the administration of Wave 1 and Wave 2.

Our primary objective is to determine whether the occurrence of these events is associated with individual-level change in support for political violence in a general population sample. To our knowledge, such a study has not been conducted previously. Our main hypothesis is that events previously linked to disinvolvement with extremist or terrorist violence [14–19], much of which is politically motivated, will be associated here with decreased support for political violence. We also hypothesize that events opposite in nature to those previously linked to disinvolvement with extremist or terrorist violence (loss of employment, for example) will be associated here with increased support for political violence. Our secondary objective is to determine whether there are patterns of (co-) occurrence for these events and assess whether such patterns are associated with support for political violence. The study is motivated by the authors’ belief that a better understanding of the reasons for change in individuals’ involvement with political violence will further efforts to prevent such violence.

## Methods

Methods for Wave 2 of this cohort survey closely followed those for Wave 1 [9]. Wave 2 was designed by the authors and administered online in English and Spanish from May 18 to June 8, 2023, by the survey research firm Ipsos [23]. The study was reviewed by the University of California Davis Institutional Review Board (protocol 1871725: exempt from full review, category 2, survey research). Before participants accessed the questionnaire, they were provided informed consent language that concluded, “[by] continuing, you are agreeing to participate in this study.” The study is reported following American Association for Public Opinion Research guidelines [24].

### Participants

Participants for Wave 1 were drawn from the Ipsos KnowledgePanel, an online research panel that has been widely used in population-based research on violence and firearm ownership [25–30]. To establish a nationally representative panel, KnowledgePanel members are recruited on an ongoing basis through address-based probability sampling using data from the US Postal Service’s Delivery Sequence File [31, 32]. Recruitment into KnowledgePanel involves repeated contact attempts, if necessary, by mail and telephone. Recruited adults in households without internet access are provided a web-enabled device and free internet service, and a modest, primarily points-based incentive program seeks to encourage participation and promote participants’ retention in KnowledgePanel over time [31, 32].

A probability-proportional-to-size procedure was used to select a study-specific sample for Wave 1. All panel members who were aged 18 years and older were eligible for selection. Invitations were sent by e-mail; automatic reminders were delivered to non-respondents by e-mail and telephone beginning 3 days later [31, 32].

The Wave 1 survey was conducted May 13 to June 2, 2022. It included a main sample, which had a completion rate of 53% and provided the study population for our initial report [9], and oversamples of firearm owners, transgender people, combat veterans, and California residents that were recruited to ensure adequate power for planned analyses. Compared with main sample nonrespondents, main sample respondents were older and more frequently white, non-Hispanic; were more often married; had higher education and income; and were less likely to be working [12].

Including the main sample and oversamples, Wave 1 comprised 12,947 respondents. Of those respondents, 11,140 (86.0%) remained active members of KnowledgePanel on Wave 2’s launch date and were invited to participate in Wave 2. (The 1807 Wave 1 respondents who were not active members of KnowledgePanel on Wave 2’s launch date left the cohort through normal attrition).

A final Wave 2 survey weight variable provided by Ipsos adjusted for the initial probability of selection into KnowledgePanel and for survey-specific nonresponse and over- or under-coverage using design weights with post-stratification raking ratio adjustments. As with the 2022 sample, the weighted 2023 sample is designed to be statistically representative of the noninstitutionalized adult population of the USA as reflected in the 2021 March supplement of the Current Population Survey [31, 32].

### Measures

Sociodemographic data were collected by Ipsos from profiles created and maintained by KnowledgePanel members. Over 3 questions, respondents were asked about the occurrence of 18 life events: “since you responded to our survey last May or June” in the first question, and “over the past year” in the second and third questions. Examples of events that had been associated with disinvolvement from extremist or terrorist violence [14–19] include “I had a child or grandchild,” “I suffered a serious illness,” “things got better for me financially,” and “I got a new job, or a better job.” We added events that were opposite to those suggested by the disinvolvement literature [9–16] and therefore expected to have opposite associations with political violence; examples include “things got worse for me financially” and “I lost my job or had my hours cut back.”

In questions concerning political violence, we used “force or violence” instead of “violence” and defined it as “physical force strong enough that it could cause pain or injury to a person.” We described political violence as “force or violence to advance an important political objective that you support.”

As in Wave 1, respondents were asked about the extent to which they considered political violence to be justified “in general” and then about justification for its use to advance specified political objectives. Example objectives include “to return Donald Trump to the presidency this year,” “to preserve an American way of life based on Western European traditions,” and “to stop police violence.” Responses for 17 objectives were collected in both waves. In Wave 1, 9 of 17 were presented to all respondents and 8 were paired, with respondents randomized for each pair to see 1 question; each respondent was presented with 13 of 17 objectives. In Wave 2, all 17 objectives were presented to all respondents.

Respondents who considered political violence to be at least sometimes justified for at least 1 of these objectives were asked about their personal willingness to engage in political violence: by type of violence (to “damage property,” “threaten or intimidate a person,” “injure a person,” “kill a person”) and by target population (examples: “an elected federal or state government official,” “a police officer,” “a person who does not share your religion”).

All respondents were asked about the likelihood of their future use of firearms in a situation where they consider political violence to be justified (examples: “I will be armed with a gun”; “I will shoot someone with a gun”).

The full text of all questions reported on here is in the Supplement (See Additional File 1).

### Implementation

Ipsos translated the questionnaire into Spanish, and interpreting services staff at UC Davis Medical Center reviewed the translation. Thirty-three KnowledgePanel members participated in a pretest of the English language version that was administered May 5–9, 2023.

Respondents were randomized 1:1 to receive response options in order from either negative to positive valence (example: from ‘do not agree’ to ‘strongly agree’) or the reverse throughout the questionnaire. Where a question presented multiple statements for respondents to consider, the order in which those statements were presented was randomized unless ordering was necessary. Logic-driving questions (those to which responses might invoke a skip pattern) included non-response prompts.

We employed unipolar response arrays without a neutral midpoint (e.g., do not agree, somewhat agree, strongly agree, very strongly agree). The literature is not in agreement on whether such midpoints should be included [33, 34]. We were persuaded by the studies reviewed by Chyung et al. [33], which suggest that such midpoints allow respondents to choose “a minimally acceptable response as soon as it is found, instead of putting effort to find an optimal response,” a behavior known as satisficing. According to those authors, satisficing is particularly common when respondents are uncomfortable with the topics of the survey or under social desirability pressures, and both conditions apply here. Our analyses focus on responses above the “somewhat” or “sometimes” level to minimize the impact of potential satisficing on the results.

### Statistical analysis

Analyses were conducted using SAS version 9.4 (SAS Institute, Inc., Cary, NC) and R, version 4.4.2 (R Foundation for Statistical Computing, Vienna, Austria).

Questions for our outcome measures had ordinal response options and were subject to non-response. We assigned integer values (“points”) to ordinal levels, with higher values indicating higher support for or willingness to engage in political violence (example: never justified = 0, somewhat justified = 1, usually justified = 2, always justified = 3). The potential range in points, aggregating across all 35 questions, was from 0 to 118.

We then used polychoric principal component analysis (PCA) [35] to identify patterns in responses to the political violence measures using the *psych* package in R [36]; polychoric correlation respects the ordinal structure of the data in estimating the correlation matrix. All complete pairs of data were used to estimate the correlation matrix, and scores for incomplete data were computed by imputing the median for missing values. The 2023 data were used to derive the factors, since each participant was presented with questions for all 35 political violence measures in 2023 but not in 2022. We retained only the first factor from the PCA, based on the observation of the scree plot (see Additional File 1, Supplement, Figure S1a) and general interpretability. Factor loadings from the first component were positively correlated with support for political violence for each of the 35 political violence measures (see Additional File 1, Supplement, Table S1).

We computed summary individual component scores for the political violence measures for each individual for 2022 and 2023, each defined as a weighted average of the individual’s responses for that year with weights determined by the loadings of the first component of the polychoric PCA on the 2023 data. We then calculated change scores as the difference between the 2 component scores.

Change scores were trimodal (see Additional File 1, Supplement, Figure S2). Due to missingness (respondents received randomized subsets of some questions in 2022), respondents with no measured change did not necessarily have a change score of zero. To describe that trimodal distribution, we therefore used K-means clustering (R, *stats* package) to categorize our respondents into groups experiencing a decreas, no change, or an increase in support for political violence.

To assist with interpreting the magnitude of change reflected in change scores, we quantified the relationship between those scores and changes in individuals’ responses to the political violence questions by categorizing the scores and determining the mean of the summed change in responses to the political violence questions for each change score category. The results are in Table S2 (see Additional File 1, Supplement).

The main analysis included all respondents in 2022 and 2023. We first assessed variation in individual component scores in 2022 and 2023 and change scores across categories of sociodemographic, political, and individual characteristics by comparing means and their 95% confidence intervals (CIs). We then assessed variation in these change scores across responses to the 18 life events. These analyses were conducted using SAS PROC SURVEYMEANS.

In multivariable analyses, we assessed whether each life event was associated with change in support for political violence in a linear regression model that accounted for complex survey weights, using SAS PROC SURVEYREG. In Model 1, the outcome was the change score; covariates included age, gender, race and ethnicity, education, income, region, marital status, work status, the presence of children in the home, political ideology, firearm ownership, and veteran status. We selected covariates based on concordance with theory and findings from prior research [9, 10]. In Model 2, we additionally adjusted for the occurrence of all other life events.

We then assessed associations among respondent subgroups of interest: men, women, respondents who thought violence was usually or always justified to advance at least 1 political objective in Wave 1, and respondents who strongly or very strongly approved of at least 1 of 11 extremist organizations or movements in Wave 1 (see Additional File 1, Supplement, Table S3). The latter 2 subgroups were included because they were found in the 2022 survey to very frequently justify and be willing to engage in political violence [12, 37].

Using SAS PROC SURVEYFREQ, we also determined the incidence of each of the 18 life events in the year between Wave 1 and Wave 2 and the frequency of respondents having experienced 0, 1, 2, 3, 4, and ≥ 5 events. Using Model 1, we then assessed the association between having experienced each specified number of life events and our outcome measures.

To determine whether there were meaningful subsets of the 18 life events, we conducted a hierarchical cluster analysis using SAS PROC VARCLUS. Hierarchical clustering is a nonparametric method with few assumptions on the input variables and more robust to outliers than parametric methods. Clusters are created by cutting the tree at a value of a distance metric chosen for the method with the number of clusters being a function of the distance metric. We evaluated metrics from the cluster summary tables for 5 different cluster solutions (see Additional File 1, Supplement, Figure S3), and the structure matrices for each solution. We selected the 3-cluster solution based on clean and discriminating cluster loadings in its structure matrix (see Additional File 1, Supplement, Table S4), the interpretability of the clusters in terms of unified underlying constructs, and an even distribution of life events in each cluster (see Additional File 1, Supplement, Table S5).

Cluster 1 events were both positive and negative and concerned the intimate partner/marriage relationship, family and extended family, employment, and illness. Cluster 2 events were negative and concerned financial status, crime and violence, mental health, and politics. Cluster 3 events were positive and concerned financial status, education, relationships with others, and social networks. We assessed the association between each cluster and change in support for political violence using the regression models described above.

In Wave 1 [12], we observed that most respondents who considered political violence to be usually or always justified were unwilling to engage in it themselves. We therefore assessed associations between life events and change scores for what we term the justification, willingness, and firearm subsets of the 35 political violence measures: 18 questions regarding justification for political violence, in general or to advance specific objectives (examples: to return Donald Trump to the presidency this year, to stop illegal immigration); 13 questions regarding willingness to engage in political violence of varying types (examples: to damage property, to kill a person) and against specified target populations (examples: a public health official, a person who does not share your religion); and 4 questions regarding future likelihood of possessing and using firearms in a situation where political violence is perceived as justified (examples: I will be armed with a gun; I will shoot someone with a gun) (see Additional File 1, Supplement, Table S1).

PCAs were performed for each subset, with subset-specific first principal components again retained for score calculation (see Additional File 1, Supplement, Figure S1 b-d). Because Models 1 and 2 yielded different estimates in the main analysis, Model 2 was used for the subset analyses.

Correspondences between change scores and mean changes in responses to the political violence questions vary with the number of questions included in the calculation, and therefore differ for the subsets (See Additional File 1, Supplement, Table S2). For example, a change score ≥ 0.6 and < 0.7 corresponds to a mean increase of 8.02 (SD 2.09) points across responses to all 35 questions—0.23 points per question, or roughly 1 point (such as from “sometimes justified” to “usually or always justified”) for 1 out of every 4 questions (see Additional File 1, Supplement, Table S2). That same change score corresponds to mean increases of 3.49 (SD 1.66) points (0.19 points per question) across responses to the 18 questions in the justified subset, 4.36 (SD 0.78) points (0.34 points per question) across responses to the 13 questions in the willingness subset, and 1.86 (SD 0.52) points (0.47 points per question) across responses to the 4 questions in the willingness subset.

## Results

Of the 11,140 panel members invited to participate as part of the main study sample, 9385 completed the survey, yielding an 84.2% completion rate. The median survey completion time was 25 min (interquartile range, 18.6 min). Non-response was < 0.5% for life event items and < 1.2% for political violence measures.

After weighting, half of the respondents (50.7%, 95% CI 49.4%, 52.1%) were female; 62.7% (95% CI 61.2%, 64.1%) were white, non-Hispanic (see Additional File 1, Supplement, Table S6). The weighted mean (standard deviation, SD) respondent age was 48.5 (25.9) years. Nonrespondents were younger than respondents (mean (SD) ages 52.5 (17.5) and 57.0 (16.5)) and less frequently male and white, non-Hispanic (see Additional File 1, Supplement, Table S7; the comparison is unweighted, as weights were not available for non-respondents).

Support for political violence decreased for 1868 respondents (19.9%, 95% CI 19.1%, 20.7%) from 2022 to 2023, increased for 1332 respondents (14.2%, 95% CI 13.5%, 14.9%) and remained unchanged for 6185 respondents (65.9%, 95% CI 64.9%, 66.9%) (see Additional File 1, Supplement, Figure S2). Group-level decreases occurred among persons ages 45–54, women, widowed persons, and political liberals; there was an increase among men (see Additional File 1, Supplement, Table S8).

Life events ranged in incidence from 0.6% (95% CI 0.5%, 0.8%) for “I was arrested or convicted of a crime, or I spent time in jail or prison” to 30.2% (95% CI 29.3%, 31.2%) for “I made some good new friends” (Table 1). Most respondents experienced multiple events; only 20.6% (95% CI 19.8%, 21.4%) experienced none (Table 1). Similar percentages of respondents experienced events in each of the 3 event clusters (range, 44.6% to 52.8%) (Table 1).

**Table 1:** Incidence of life events, mean scores for support of political violence, and mean change scores

Life Event	Unweighted *n*	Weighted % (95% CI)	2022 score Mean (95% CI)	2023 score Mean (95% CI)	Change score Mean (95% CI)
I had a child or a grandchild^*^					
No	8444	90.2 (89.6, 90.8)	−0.01 (−0.04, 0.01)	−0.02 (−0.05, 0.01)	−0.01 (−0.03, 0.02)
Yes	913	9.8 (9.2, 10.4)	0.17 (0.06, 0.27)	0.15 (0.03, 0.26)	−0.02 (−0.12, 0.08)
I started a new romantic relationship, or an existing relationship grew stronger^*^					
No	8337	89.2 (88.5, 89.8)	−0.01 (−0.03, 0.02)	−0.03 (−0.05, 0.00)	−0.02 (−0.05, 0.01)
Yes	1014	10.8 (10.2, 11.5)	0.09 (0.01, 0.16)	0.15 (0.06, 0.24)	0.06 (−0.02, 0.15)
I got married or engaged^*^					
No	9013	96.4 (96.0, 96.8)	−0.01 (−0.03, 0.02)	−0.02 (−0.05, 0.01)	−0.01 (−0.04, 0.01)
Yes	339	3.6 (3.2, 4.0)	0.20 (0.04, 0.36)	0.32 (0.14, 0.51)	0.13 (−0.04, 0.29)
I got a new job, or a better job^*^					
No	8493	90.8 (90.2, 91.4)	0.00 (−0.03, 0.02)	−0.01 (−0.04, 0.02)	−0.01 (−0.04, 0.01)
Yes	860	9.2 (8.6, 9.8)	0.06 (−0.03, 0.14)	0.08 (−0.02, 0.17)	0.02 (−0.07, 0.11)
I lost my job or had my hours cut back^*^					
No	8860	94.7 (94.3, 95.2)	−0.01 (−0.03, 0.02)	−0.03 (−0.06, 0.00)	−0.02 (−0.05, 0.00)
Yes	495	5.3 (4.8, 5.7)	0.16 (0.03, 0.29)	0.36 (0.21, 0.51)	**0.20 (0.06**,** 0.34)**^**§**^
I suffered a serious illness^*^					
No	8522	91.1 (90.5, 91.7)	−0.01 (−0.04, 0.02)	−0.02 (−0.05, 0.01)	−0.01 (−0.04, 0.01)
Yes	831	8.9 (8.3, 9.5)	0.17 (0.07, 0.27)	0.25 (0.13, 0.36)	0.08 (−0.03, 0.18)
My partner, a close family member, or a close friend suffered a serious illness or died^*^					
No	7380	78.8 (78.0, 79.7)	−0.01 (−0.04, 0.02)	−0.02 (−0.06, 0.01)	−0.02 (−0.05, 0.01)
Yes	1980	21.2 (20.3, 22.0)	0.06 (−0.01, 0.12)	0.09 (0.02, 0.16)	0.03 (−0.02, 0.09)
Things got worse for me financially^†^					
No	7471	79.9 (79.1, 80.7)	−0.02 (−0.05, 0.01)	−0.04 (−0.07, −0.01)	−0.02 (−0.05, 0.01)
Yes	1876	20.1 (19.3, 20.9)	0.11 (0.05, 0.17)	0.13 (0.07, 0.20)	0.03 (−0.03, 0.09)
Things improved for me financially^‡^					
No	6643	71.0 (70.1, 71.9)	0.00 (−0.03, 0.03)	0.01 (−0.02, 0.04)	0.01 (−0.02, 0.04)
Yes	2711	29.0 (28.1, 29.9)	0.01 (−0.04, 0.06)	−0.03 (−0.09, 0.02)	−0.04 (−0.09, 0.01)
I started a new educational activity or completed one^‡^					
No	8624	92.2 (91.7, 92.7)	0.00 (−0.02, 0.03)	−0.02 (−0.05, 0.01)	−0.02 (−0.05, 0.01)
Yes	730	7.8 (7.3, 8.3)	0.01 (−0.08, 0.10)	0.11 (0.01, 0.21)	**0.10 (0.01**,** 0.19)**
I started a new social or community service activity^‡^					
No	8542	91.3 (90.7, 91.9)	0.00 (−0.03, 0.03)	−0.02 (−0.05, 0.01)	−0.02 (−0.04, 0.01)
Yes	812	8.7 (8.1, 9.3)	0.04 (−0.05, 0.13)	0.13 (0.02, 0.23)	0.09 (−0.01, 0.19)
I made some good new friends^‡^					
No	6529	69.8 (68.8, 70.7)	−0.02 (−0.05, 0.01)	−0.05 (−0.08, −0.01)	−0.02 (−0.05, 0.01)
Yes	2829	30.2 (29.3, 31.2)	0.06 (0.01, 0.12)	0.09 (0.04, 0.15)	0.03 (−0.02, 0.08)
I got burned out^†^					
No	7610	81.4 (80.6, 82.1)	0.00 (−0.03, 0.03)	−0.02 (−0.06, 0.01)	−0.02 (−0.05, 0.01)
Yes	1744	18.6 (17.9, 19.4)	0.04 (−0.02, 0.09)	0.07 (0.01, 0.13)	0.03 (−0.02, 0.09)
I was arrested or convicted of a crime, or I spent time in jail or prison^†^					
No	9295	99.4 (99.2, 99.5)	0.00 (−0.03, 0.02)	−0.02 (−0.04, 0.01)	−0.01 (−0.04, 0.01)
Yes	57	0.6 (0.5, 0.8)	0.80 (0.29, 1.31)	1.43 (0.83, 2.03)	**0.63 (0.04**,** 1.21)**
I gave up on politics^†^					
No	7557	80.9 (80.1, 81.7)	0.00 (−0.03, 0.03)	−0.02 (−0.06, 0.01)	−0.02 (−0.05, 0.00)
Yes	1787	19.1 (18.3, 19.9)	0.03 (−0.03, 0.09)	0.09 (0.02, 0.16)	0.06 (0.00, 0.13)
My political beliefs changed a lot^†^					
No	8785	94.0 (93.5, 94.4)	−0.01 (−0.04, 0.01)	−0.02 (−0.05, 0.01)	−0.01 (−0.03, 0.02)
Yes	565	6.0 (5.6, 6.5)	0.28 (0.15, 0.42)	0.28 (0.15, 0.41)	−0.01 (−0.15, 0.13)
I decided there was too much violence in my life^†^					
No	8985	96.1 (95.7, 96.5)	−0.01 (−0.04, 0.01)	−0.03 (−0.06, 0.00)	−0.01 (−0.04, 0.01)
Yes	362	3.9 (3.5, 4.3)	0.47 (0.25, 0.69)	0.61 (0.39, 0.84)	0.14 (−0.03, 0.32)
I had a positive experience with someone I thought was my enemy^‡^					
No	8844	94.6 (94.2, 95.1)	−0.01 (−0.04, 0.02)	−0.03 (−0.05, 0.00)	−0.02 (−0.04, 0.01)
Yes	502	5.4 (4.9, 5.8)	0.23 (0.08, 0.38)	0.38 (0.21, 0.55)	**0.15 (0.02**,** 0.29)**
**Number of life events**					
0	1932	20.6 (19.8, 21.4)	−0.02 (−0.08, 0.04)	−0.07 (−0.13, 0.00)	−0.05 (−0.11, 0.01)
1	2140	22.8 (22.0, 23.7)	−0.05 (−0.09, −0.01)	−0.10 (−0.15, −0.05)	−0.05 (−0.10, 0.00)
2	1959	20.9 (20.1, 21.7)	−0.04 (−0.10, 0.01)	−0.04 (−0.10, 0.02)	0.00 (−0.05, 0.05)
3	1449	15.5 (14.7, 16.2)	0.01 (−0.06, 0.08)	−0.04 (−0.11, 0.03)	−0.05 (−0.12, 0.01)
4	884	9.4 (8.8, 10.0)	0.03 (−0.06, 0.13)	0.05 (−0.04, 0.14)	0.02 (−0.06, 0.10)
≥ 5	1011	10.8 (10.2, 11.4)	0.17 (0.09, 0.26)	0.31 (0.21, 0.41)	**0.14 (0.05**,** 0.23)**
**Event cluster**					
Cluster 1^*^	4096	44.6 (43.2, 46.0)	0.04 (0.00, 0.08)	0.06 (0.01, 0.10)	0.01 (−0.02, 0.05)
Cluster 2^†^	4113	46.3 (44.9, 47.7)	0.05 (0.01, 0.09)	0.07 (0.03, 0.11)	0.02 (−0.01, 0.06)
Cluster 3^‡^	4798	52.8 (51.4, 54.2)	0.01 (−0.03, 0.05)	0.02 (−0.02, 0.06)	0.01 (−0.03, 0.05)

Information on the relationship between change scores and change in responses to political violence questions is given in Table S2 (see Additional File 1, Supplement). For reference, a change score mean of 0.63 (the high score in this table) corresponds to a mean change of 0.23 points per question. A change score mean of −0.05 (the low score in this table) corresponds to a mean change of −0.06 points per question

^*^ Cluster 1 events were both positive and negative and concerned the partner/marriage relationship, family and extended family, employment, and health status

^†^ Cluster 2 events were negative and concerned financial status, crime and violence, mental health, and politics

^‡^ Cluster 3 events were positive and concerned financial status, education, relationships with others, and social networks

^§^ Statistically significant change scores (*P* < 0.05) are in bold font

### Life events and change scores

When considered individually in unadjusted models, no life events were associated with a decrease in support for political violence (Table 1). Four events were associated with an increase: “I lost my job or had my hours cut back” (change score 0.20, 95% CI 0.06, 0.34), “I started a new educational activity or completed one” (change score 0.10, 95% CI 0.01, 0.19), “I was arrested or convicted of a crime, or I spent time in jail or prison” (change score 0.63, 95% CI 0.04, 1.21), and “I had a positive experience with someone I thought was my enemy” (change score 0.15, 95% CI 0.02, 0.29). When considered cumulatively, only the occurrence of ≥ 5 events was associated with a change in support for political violence; this was an increase (change score 0.14, 95% CI 0.05, 0.23). Respondents who experienced ≥ 5 events were far more likely than other to have experienced job loss (prevalence ratio 10.5), arrest or incarceration (prevalence ratio 23.4), or both those events (prevalence ratio 28.5). None of the event clusters was associated with a change in support for political violence (Table 1).

**Fig. 1:**
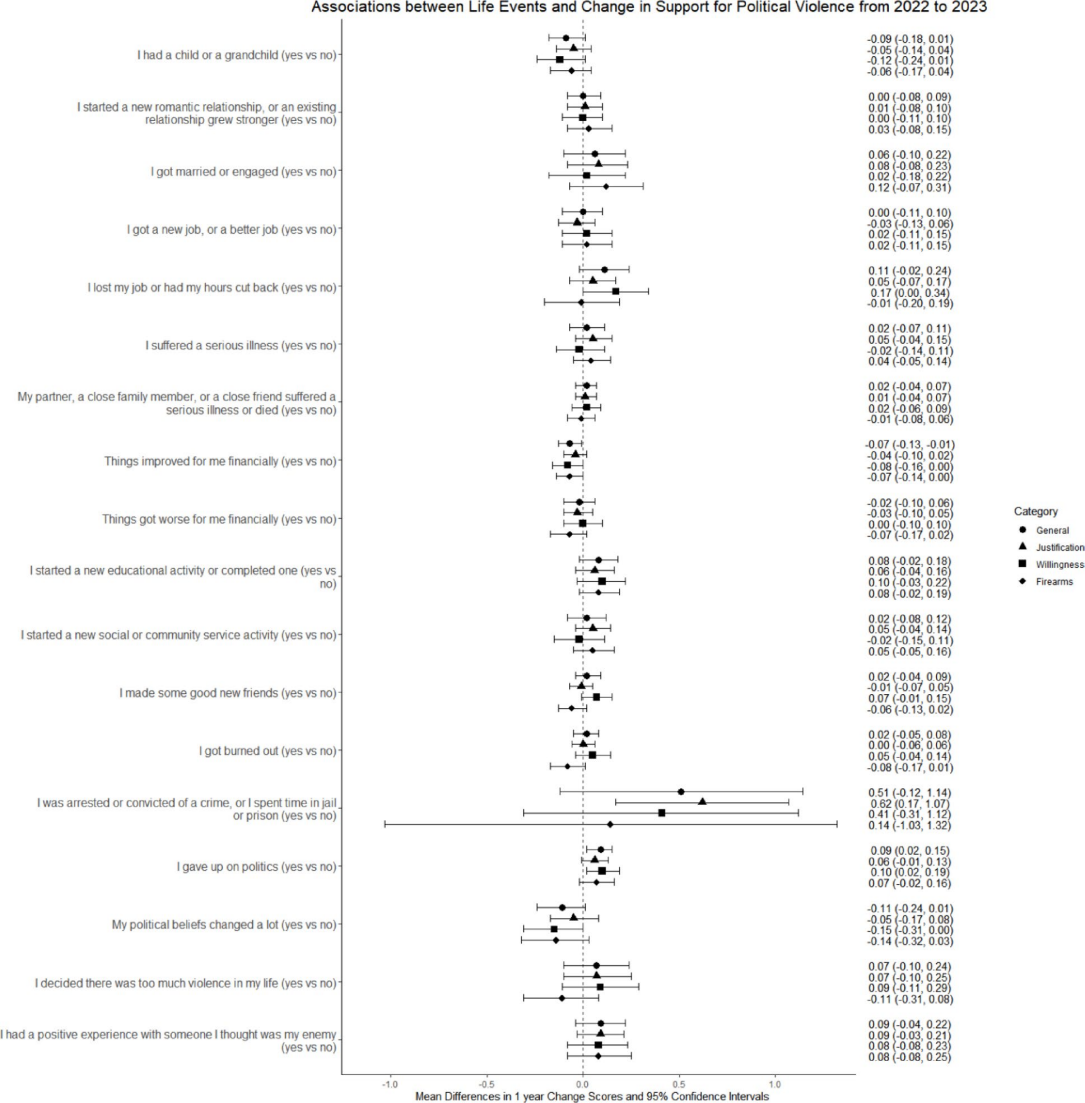
Life events and change in support for political violence, including subsets* of political violence measures. Information on the relationship between change scores and change in responses to political violence questions is given in Table S2 (see Additional File 1, Supplement). This relationship varies with the number of questions included in the calculation and therefore differs across question subsets.* Justification (political violence is usually or always justified), willingness (very or completely willing to engage in political violence), firearm (very or extremely likely to use firearms)

Table 2; Fig. 1 display the adjusted mean difference (aMD) in change scores between respondents experiencing and not experiencing each life event. In Model 2, “I gave up on politics” was associated with increased support for political violence (aMD 0.09, 95% CI 0.02, 0.15), and “things improved for me financially” was associated with decreased support (aMD − 0.07, 95% CI −0.13, −0.01). Arrest or incarceration (aMD 0.51, 95% CI −0.12, 1.14) was associated with a large increase that was not statistically significant. The occurrence of ≥ 5 events was again associated with an increase in support for political violence, and there was an increase associated with the negative life events in Cluster 2.

**Table 2:** Life events and adjusted mean differences in change scores

Life event	Model 1, Adjusted Mean Difference (95% CI)^*^	Model 2, Adjusted Mean Difference (95% CI) ^†^
I had a child or a grandchild (yes vs. no)	−0.02 (−0.13, 0.09)	−0.09 (−0.18, 0.01)
I started a new romantic relationship, or an existing relationship grew stronger (yes vs. no)	0.06 (−0.03, 0.16)	0.00 (−0.08, 0.09)
I got married or engaged (yes vs. no)	0.15 (−0.03, 0.33)	0.06 (−0.10, 0.22)
I got a new job, or a better job (yes vs. no)	0.04 (−0.06, 0.15)	0.00 (−0.11, 0.10)
I lost my job or had my hours cut back (yes vs. no)	**0.18 (0.05**,** 0.32)**^**‡**^	0.11 (−0.02, 0.24)
I suffered a serious illness (yes vs. no)	0.10 (−0.01, 0.22)	0.02 (−0.07, 0.11)
My partner, a close family member, or a close friend suffered a serious illness or died (yes vs. no)	0.05 (−0.02, 0.11)	0.02 (−0.04, 0.07)
Things improved for me financially (yes vs. no)	−0.04 (−0.10, 0.02)	**−0.07 (−0.13**,** −0.01)**
Things got worse for me financially (yes vs. no)	0.05 (−0.02, 0.12)	−0.02 (−0.10, 0.06)
I started a new educational activity or completed one (yes vs. no)	**0.12 (0.02**,** 0.22)**	0.08 (−0.02, 0.18)
I started a new social or community service activity (yes vs. no)	0.09 (−0.02, 0.20)	0.02 (−0.08, 0.12)
I made some good new friends (yes vs. no)	0.04 (−0.02, 0.11)	0.02 (−0.04, 0.09)
I got burned out (yes vs. no)	0.06 (−0.00, 0.13)	0.02 (−0.05, 0.08)
I was arrested or convicted of a crime, or I spent time in jail or prison (yes vs. no)	0.59 (−0.00, 1.19)	0.51 (−0.12, 1.14)
I gave up on politics (yes vs. no)	**0.10 (0.02**,** 0.17)**	**0.09 (0.02**,** 0.15)**
My political beliefs changed a lot (yes vs. no)	−0.01 (−0.15, 0.12)	−0.11 (−0.24, 0.01)
I decided there was too much violence in my life (yes vs. no)	0.18 (−0.01, 0.36)	0.07 (−0.10, 0.24)
I had a positive experience with someone I thought was my enemy (yes vs. no)	**0.15 (0.01**,** 0.29)**	0.09 (−0.04, 0.22)
**Number of life events**		
0	Reference	N/A
1	0.00 (−0.07, 0.08)	N/A
2	0.06 (−0.02, 0.14)	N/A
3	0.00 (−0.08, 0.09)	N/A
4	0.09 (−0.02, 0.19)	N/A
≥ 5	**0.19 (0.09**,** 0.30)**	N/A
**Event cluster** ^**§**^		
Cluster 1 (yes vs. no)	0.04 (−0.02, 0.09)	0.03 (−0.03, 0.08)^¶^
Cluster 2 (yes vs. no)	**0.06 (0.01**,** 0.12)**	**0.06 (0.00**,** 0.11)**
Cluster 3 (yes vs. no)	0.03 (−0.02, 0.08)	0.02 (−0.03, 0.08)

Information on the relationship between change scores and change in responses to political violence questions is given in Table S2 (see Additional File 1, Supplement). For reference, an adjusted mean difference in change scores of 0.19 (the largest positive difference in this table) corresponds to a mean change of 0.02 points per question. A mean difference in change scores of −0.09 (the largest negative difference in this table) corresponds to a mean change of −0.06 points per question

^*^ Results are from a linear regression model which accounts for complex survey weights. Each life event is included in a separate model. Models are adjusted for age, gender, race and ethnicity, education, income, region, marital status, work status, having children in the household, political ideology, firearm ownership, and veteran status

^**†**^ Additionally adjusted for all other life events

^‡^ Statistically significant change scores (*P* < 0.05) are in bold font

^§^ Cluster 1 events were both positive and negative and concerned the partner/marriage relationship, family and extended family, employment, and health status. Cluster 2 events were negative and concerned financial status, crime and violence, mental health, and politics. Cluster 3 events were positive and concerned financial status, education, relationships with others, and social networks

^¶^ Additionally adjusted for all other clusters

### Life events and change scores for outcome subsets

Associations between the occurrence of individual events and the justification, willingness, and firearm subsets of the political violence outcome measures are presented in Table S9 (see Additional File 1, Supplement) and Fig. 1. An improved financial situation was associated with decreased willingness to engage in political violence (aMD − 0.08, 95% CI −0.16, 0.00) and a decreased expectation of firearm use (aMD − 0.07, 95% CI −0.14, 0.00); arrest and incarceration were associated with an increase in justification for violence (aMD 0.62, 95% CI 0.17, 1.07) and a large but statistically non-significant increase in willingness to engage in it (aMD 0.41, 95% CI −0.31, 1.12); giving up on politics was associated with an increased willingness to engage in political violence (aMD 0.10, 95% CI 0.02, 0.19). Experiencing ≥ 5 events was associated with increases in justification for and willingness to engage in political violence (justification, aMD 0.14, 95% CI 0.04, 0.24; willingness, aMD 0.25, 95% CI 0.11, 0.38). Experiencing the negative events in Cluster 2 was associated with an increased willingness to engage in political violence (aMD 0.10, 95% CI 0.03, 0.17) but a decreased expectation of firearm use (aMD − 0.07, 95% CI −0.13, 0.00).

### Life events and change scores for respondent subsets

**Fig. 2:**
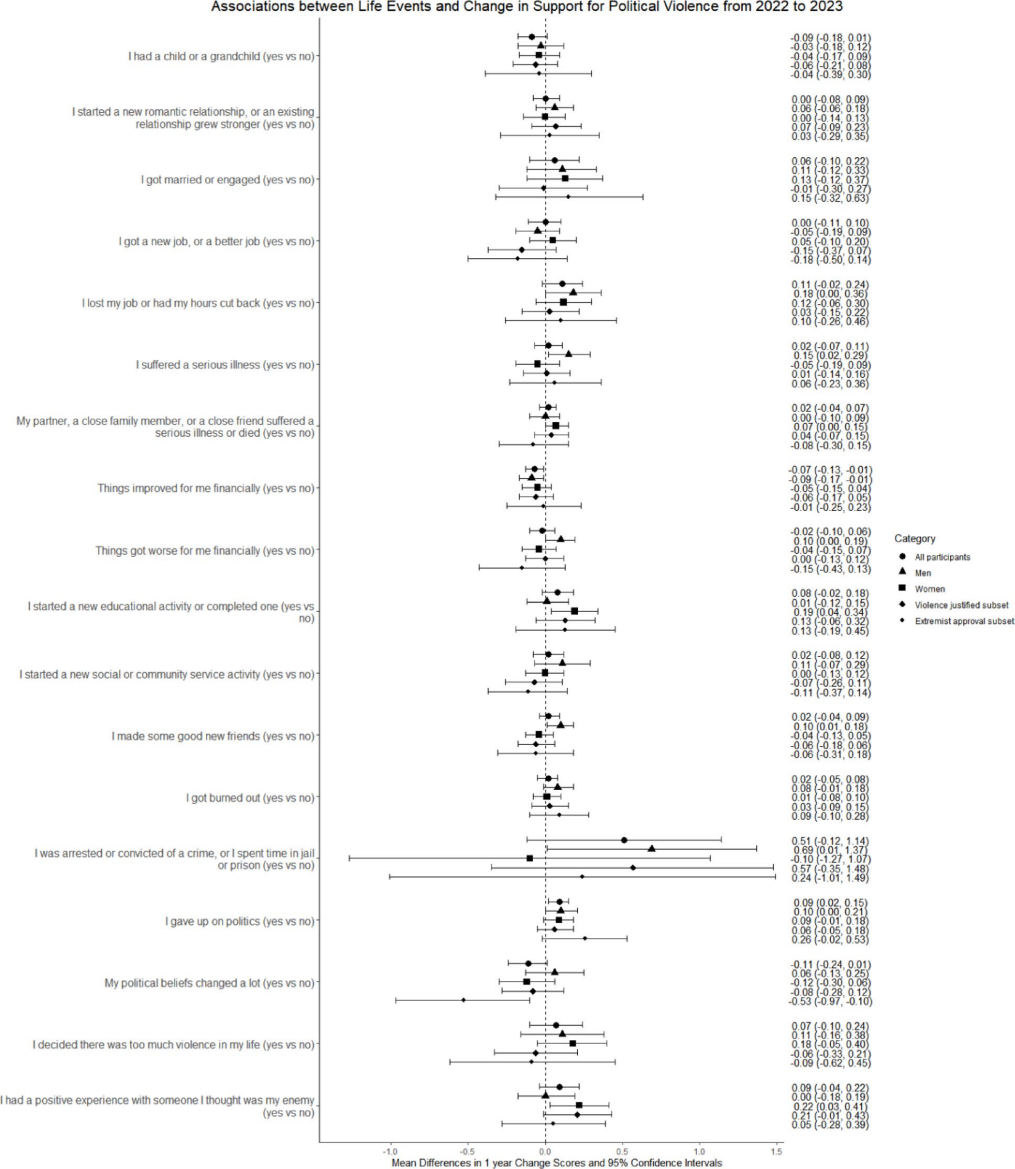
Life events and change in support for political violence among subsets of respondents*. Information on the relationship between change scores and change in responses to political violence questions is given in Table S2 (see Additional File 1, Supplement). For reference, an adjusted mean difference in change scores of 0.69 (the largest positive difference in this figure) corresponds to a mean change of 0.23 points per question. A mean difference in change scores of −0.12 (the largest negative difference in this figure) corresponds to a mean change of −0.11 points per question. * Subsets are men, women, respondents who indicated violence was usually or always justified to advance at least 1 political objective in 2022, and respondents who strongly or very strongly approved of at least one extremist organization/movement in 2022

Tables 3, 4 and 5; Fig. 2 present results for subgroups of respondents. No life event was associated with change among both men and women when they were analyzed separately (Table 3; Fig. 2). In Model 2, women’s support for political violence increased in association with starting “a new educational activity” (aMD 0.19, 95% CI 0.04, 0.34) and having “a positive experience with someone I thought was my enemy” (aMD 0.22, 95% CI 0.03, 0.41). Among men, support increased in association with making “some good new friends” (aMD 0.11, 95% CI 0.02, 0.19) and being arrested or incarcerated (aMD 0.75, 95% CI 0.06, 1.45) and decreased in association with an improved financial situation (aMD − 0.09, 95% CI −0.18, −0.01). Experiencing ≥ 5 events was associated with similar increases in support among both women and men (women, aMD 0.21, 95% CI 0.07, 0.35; men, aMD 0.20, 95% CI 0.04, 0.36), and experiencing Cluster 2 events was associated with increased support among men (aMD 0.08, 95% CI 0.01, 0.16).

**Table 3:** Life events and adjusted mean differences in change scores, by gender

Life event	Model 1, Adjusted Mean Difference (95% CI)^*^		Model 2, Adjusted Mean Difference (95% CI)^†^	
	Among men (*n* = 5340)	**Among women** (*n* = 3866)	Among men (*n* = 5340)	Among women (*n* = 3866)
I had a child or a grandchild (yes vs. no)	−0.03 (−0.18, 0.12)	0.00 (−0.16, 0.16)	−0.12 (−0.26, 0.01)	−0.04 (−0.17, 0.09)
I started a new romantic relationship, or an existing relationship grew stronger (yes vs. no)	0.06 (−0.06, 0.18)	0.07 (−0.07, 0.22)	0.02 (−0.10, 0.13)	0.00 (−0.14, 0.13)
I got married or engaged (yes vs. no)	0.11 (−0.12, 0.33)	0.21 (−0.08, 0.50)	0.05 (−0.17, 0.27)	0.13 (−0.12, 0.37)
I got a new job, or a better job (yes vs. no)	−0.05 (−0.19, 0.09)	0.11 (−0.04, 0.26)	−0.09 (−0.23, 0.06)	0.05 (−0.10, 0.20)
I lost my job or had my hours cut back (yes vs. no)	**0.18 (0.00**,** 0.36)**^**‡**^	0.20 (−0.02, 0.42)	0.10 (−0.09, 0.29)	0.12 (−0.06, 0.30)
I suffered a serious illness (yes vs. no)	**0.15 (0.02**,** 0.29)**	0.06 (−0.11, 0.23)	0.10 (−0.02, 0.22)	−0.05 (−0.19, 0.09)
My partner, a close family member, or a close friend suffered a serious illness or died (yes vs. no)	0.00 (−0.10, 0.09)	**0.09 (0.00**,** 0.18)**	−0.05 (−0.15, 0.04)	0.07 (0.00, 0.15)
Things improved for me financially (yes vs. no)	**−0.09 (−0.17**,** −0.01)**	−0.01 (−0.09, 0.08)	**−0.09 (−0.18**,** −0.01)**	−0.05 (−0.15, 0.04)
Things got worse for me financially (yes vs. no)	0.10 (0.00, 0.19)	0.02 (−0.08, 0.12)	0.02 (−0.09, 0.13)	−0.04 (−0.15, 0.07)
I started a new educational activity or completed one (yes vs. no)	0.01 (−0.12, 0.15)	**0.23 (0.09**,** 0.38)**	−0.02 (−0.16, 0.11)	**0.19 (0.04**,** 0.34)**
I started a new social or community service activity (yes vs. no)	0.11 (−0.07, 0.29)	0.06 (−0.07, 0.19)	0.05 (−0.13, 0.22)	0.00 (−0.13, 0.12)
I made some good new friends (yes vs. no)	**0.10 (0.01**,** 0.18)**	0.00 (−0.09, 0.08)	**0.11 (0.02**,** 0.19)**	−0.04 (−0.13, 0.05)
I got burned out (yes vs. no)	0.08 (−0.01, 0.18)	0.06 (−0.04, 0.16)	0.05 (−0.04, 0.14)	0.01 (−0.08, 0.10)
I was arrested or convicted of a crime, or I spent time in jail or prison (yes vs. no)	**0.69 (0.01**,** 1.37)**	0.34 (−0.87, 1.54)	**0.75 (0.06**,** 1.45)**	−0.10 (−1.27, 1.07)
I gave up on politics (yes vs. no)	0.10 (0.00, 0.21)	0.08 (−0.02, 0.19)	0.07 (−0.02, 0.17)	0.09 (−0.01, 0.18)
My political beliefs changed a lot (yes vs. no)	0.06 (−0.13, 0.25)	−0.05 (−0.24, 0.14)	−0.04 (−0.20, 0.12)	−0.12 (−0.30, 0.06)
I decided there was too much violence in my life (yes vs. no)	0.11 (−0.16, 0.38)	0.26 (0.00, 0.52)	−0.07 (−0.33, 0.20)	0.18 (−0.05, 0.40)
I had a positive experience with someone I thought was my enemy (yes vs. no)	0.00 (−0.18, 0.19)	**0.26 (0.06**,** 0.47)**	−0.10 (−0.27, 0.07)	**0.22 (0.03**,** 0.41)**
**Number of life events**				
0	Reference	Reference	N/A	N/A
1	−0.06 (−0.16, 0.04)	0.07 (−0.04, 0.18)	N/A	N/A
2	0.02 (−0.09, 0.13)	0.10 (−0.01, 0.20)	N/A	N/A
3	−0.01 (−0.14, 0.12)	0.02 (−0.10, 0.14)	N/A	N/A
4	0.08 (−0.06, 0.22)	0.12 (−0.03, 0.26)	N/A	N/A
≥ 5	**0.20 (0.04**,** 0.36)**	**0.21 (0.07**,** 0.35)**	N/A	N/A
**Event cluster** ^§^				
**Cluster 1** (yes vs. no)	0.01 (−0.06, 0.09)	0.06 (−0.02, 0.13)	0.00 (−0.08, 0.07)	0.05 (−0.03, 0.12)^¶^
**Cluster 2** (yes vs. no)	**0.08 (0.01**,** 0.16)**	0.05 (−0.03, 0.13)	**0.08 (0.01**,** 0.16)**	0.04 (−0.03, 0.12)
**Cluster 3** (yes vs. no)	0.01 (−0.07, 0.09)	0.04 (−0.03, 0.11)	0.01 (−0.07, 0.08)	0.03 (−0.04, 0.10)

Information on the relationship between change scores and change in responses to political violence questions is given in Table S2 (see Additional File 1, Supplement). For reference, an adjusted mean difference in change scores of 0.69 (the largest positive difference in this table) corresponds to a mean change of 0.23 points per question. A mean difference in change scores of −0.12 (the largest negative difference in this table) corresponds to a mean change of −0.11 points per question

^*^ Results are from a linear regression model which accounts for complex survey weights. Each life event is included in a separate model. Models are adjusted for age, gender, race and ethnicity, education, income, region, marital status, work status, having children in the household, political ideology, firearm ownership, and veteran status

^**†**^ Additionally adjusted for all other life events

^‡^ Statistically significant change scores (*P* < 0.05) are in bold font

^§^ Cluster 1 events were both positive and negative and concerned the partner/marriage relationship, family and extended family, employment, and health status. Cluster 2 events were negative and concerned financial status, crime and violence, mental health, and politics. Cluster 3 events were positive and concerned financial status, education, relationships with others, and social networks

^¶^ Additionally adjusted for all other clusters

**Table 4:** Life events and adjusted mean differences in change scores, among respondents who justified violence

Life event	Model 1, Adjusted Mean Difference (95% CI)^*^	Model 2, Adjusted Mean Difference (95% CI)^†^
I had a child or a grandchild (yes vs. no)	−0.04 (−0.22, 0.15)	−0.06 (−0.21, 0.08)
I started a new romantic relationship, or an existing relationship grew stronger (yes vs. no)	0.09 (−0.09, 0.27)	0.07 (−0.09, 0.23)
I got married or engaged (yes vs. no)	0.09 (−0.24, 0.42)	−0.01 (−0.30, 0.27)
I got a new job, or a better job (yes vs. no)	−0.10 (−0.32, 0.12)	−0.15 (−0.37, 0.07)
I lost my job or had my hours cut back (yes vs. no)	0.12 (−0.11, 0.35)	0.03 (−0.15, 0.22)
I suffered a serious illness (yes vs. no)	0.06 (−0.12, 0.25)	0.01 (−0.14, 0.16)
My partner, a close family member, or a close friend suffered a serious illness or died (yes vs. no)	0.06 (−0.06, 0.18)	0.04 (−0.07, 0.15)
Things improved for me financially (yes vs. no)	−0.06 (−0.18, 0.05)	−0.06 (−0.17, 0.05)
Things got worse for me financially (yes vs. no)	0.04 (−0.08, 0.16)	0.00 (−0.13, 0.12)
I started a new educational activity or completed one (yes vs. no)	0.16 (−0.05, 0.37)	0.13 (−0.06, 0.32)
I started a new social or community service activity (yes vs. no)	0.00 (−0.22, 0.21)	−0.07 (−0.26, 0.11)
I made some good new friends (yes vs. no)	−0.04 (−0.15, 0.07)	−0.06 (−0.18, 0.06)
I got burned out (yes vs. no)	0.10 (−0.04, 0.23)	0.03 (−0.09, 0.15)
I was arrested or convicted of a crime, or I spent time in jail or prison (yes vs. no)	0.52 (−0.36, 1.40)	0.57 (−0.35, 1.48)
I gave up on politics (yes vs. no)	0.09 (−0.04, 0.22)	0.06 (−0.05, 0.18)
My political beliefs changed a lot (yes vs. no)	0.00 (−0.23, 0.23)	−0.08 (−0.28, 0.12)
I decided there was too much violence in my life (yes vs. no)	0.04 (−0.25, 0.32)	−0.06 (−0.33, 0.21)
I had a positive experience with someone I thought was my enemy (yes vs. no)	0.23 (−0.03, 0.50)	0.21 (−0.01, 0.43)
**Number of life events**		
0	Reference	N/A
1	−0.04 (−0.18, 0.09)	N/A
2	0.09 (−0.06, 0.24)	N/A
3	−0.09 (−0.27, 0.08)	N/A
4	−0.02 (−0.21, 0.17)	N/A
≥ 5	**0.22 (0.02**,** 0.41)**^**‡**^	N/A
**Event cluster** ^§^		
Cluster 1 (yes vs. no)	0.06 (−0.04, 0.16)	0.05 (−0.05, 0.15)^¶^
Cluster 2 (yes vs. no)	0.09 (−0.01, 0.19)	0.09 (−0.01, 0.18)
Cluster 3 (yes vs. no)	0.00 (−0.09, 0.10)	−0.01 (−0.10, 0.08)

Information on the relationship between change scores and change in responses to political violence questions is given in Table S2 (see Additional File 1, Supplement). For reference, an adjusted mean difference in change scores of 0.57 (the largest positive difference in this table) corresponds to a mean change of 0.20 points per question. A mean difference in change scores of −0.15 (the largest negative difference in this table) corresponds to a mean change of −0.11 points per question

^*^ Results are from a linear regression model which accounts for complex survey weights. Each life event is included in a separate model. Models are adjusted for age, gender, race and ethnicity, education, income, region, marital status, work status, having children in the household, political ideology, firearm ownership, and veteran status

^**†**^ Additionally adjusted for all other life events

^‡^ Statistically significant change scores (*P* < 0.05) are in bold font

^§^ Cluster 1 events were both positive and negative and concerned the partner/marriage relationship, family and extended family, employment, and health status. Cluster 2 events were negative and concerned financial status, crime and violence, mental health, and politics. Cluster 3 events were positive and concerned financial status, education, relationships with others, and social networks

^¶^ Additionally adjusted for all other clusters

Among respondents who reported in Wave 1 that they thought violence was usually or always justified to advance at least 1 political objective (Table 4; Fig. 2), no life event was associated with change in support for political violence, but starting “a new educational activity,” being arrested or incarcerated, and having “a positive experience with someone I thought was my enemy” were associated with large positive change scores that were not statistically significant. Experiencing ≥ 5 events was again associated with increased support for political violence (aMD 0.22, 95% CI 0.02, 0.41).

**Table 5:** Life events and adjusted mean differences in change scores among respondents who approved of an extremist organization

Life event	Among all extremist organizations (*n* = 1105)		Among left-wing extremist organizations (*n* = 452)		Among right-wing extremist organizations (*n* = 767)	
	Model 1, Adjusted Mean Difference (95% CI)^*^	Model 2, Adjusted Mean Difference (95% CI)^†^	Model 1, Adjusted Mean Difference (95% CI)^*^	Model 2, Adjusted Mean Difference (95% CI)^†^	Model 1, Adjusted Mean Difference (95% CI)^*^	Model 2, Adjusted Mean Difference (95% CI)^†^
I had a child or a grandchild (yes vs. no)	−0.04 (−0.45, 0.38)	−0.04 (−0.39, 0.30)	−0.02 (−0.66, 0.62)	−0.14 (−0.63, 0.34)	−0.01 (−0.44, 0.42)	−0.03 (−0.42, 0.37)
I started a new romantic relationship, or an existing relationship grew stronger (yes vs. no)	0.08 (−0.26, 0.43)	0.03 (−0.29, 0.35)	0.16 (−0.27, 0.58)	0.09 (−0.28, 0.46)	0.17 (−0.32, 0.65)	0.13 (−0.37, 0.63)
I got married or engaged (yes vs. no)	0.18 (−0.40, 0.75)	0.15 (−0.32, 0.63)	0.61 (−0.15, 1.37)	**0.70 (0.12**,** 1.29)**	0.10 (−0.62, 0.82)	−0.01 (−0.67, 0.66)
I got a new job, or a better job (yes vs. no)	−0.10 (−0.42, 0.21)	−0.18 (−0.50, 0.14)	0.16 (−0.22, 0.55)	0.12 (−0.23, 0.47)	−0.04 (−0.49, 0.40)	−0.22 (−0.66, 0.22)
I lost my job or had my hours cut back (yes vs. no)	0.04 (−0.40, 0.48)	0.10 (−0.26, 0.46)	−0.16 (−0.81, 0.49)	−0.18 (−0.72, 0.35)	0.10 (−0.41, 0.61)	0.14 (−0.32, 0.60)
I suffered a serious illness (yes vs. no)	0.08 (−0.30, 0.46)	0.06 (−0.23, 0.36)	−0.01 (−0.59, 0.58)	−0.28 (−0.72, 0.16)	0.11 (−0.34, 0.56)	0.03 (−0.37, 0.42)
My partner, a close family member, or a close friend suffered a serious illness or died (yes vs. no)	−0.06 (−0.32, 0.21)	−0.08 (−0.30, 0.15)	0.13 (−0.23, 0.50)	0.20 (−0.12, 0.51)	−0.07 (−0.41, 0.28)	−0.09 (−0.39, 0.21)
Things improved for me financially (yes vs. no)	−0.03 (−0.24, 0.19)	−0.01 (−0.25, 0.23)	0.04 (−0.21, 0.29)	−0.02 (−0.32, 0.28)	−0.05 (−0.35, 0.25)	−0.08 (−0.41, 0.25)
Things got worse for me financially (yes vs. no)	−0.12 (−0.35, 0.12)	−0.15 (−0.43, 0.13)	−0.10 (−0.39, 0.19)	−0.10 (−0.44, 0.25)	−0.18 (−0.49, 0.14)	−0.23 (−0.60, 0.14)
I started a new educational activity or completed one (yes vs. no)	0.10 (−0.21, 0.41)	0.13 (−0.19, 0.45)	0.11 (−0.24, 0.46)	−0.02 (−0.37, 0.33)	0.14 (−0.37, 0.66)	0.30 (−0.29, 0.88)
I started a new social or community service activity (yes vs. no)	−0.16 (−0.43, 0.12)	−0.11 (−0.37, 0.14)	−0.10 (−0.48, 0.28)	0.01 (−0.28, 0.29)	−0.12 (−0.52, 0.28)	−0.14 (−0.55, 0.28)
I made some good new friends (yes vs. no)	−0.12 (−0.34, 0.10)	−0.06 (−0.31, 0.18)	−0.15 (−0.47, 0.17)	−0.18 (−0.50, 0.14)	−0.12 (−0.41, 0.17)	−0.07 (−0.40, 0.25)
I got burned out (yes vs. no)	0.06 (−0.16, 0.28)	0.09 (−0.10, 0.28)	−0.03 (−0.33, 0.27)	−0.06 (−0.31, 0.20)	−0.07 (−0.40, 0.26)	−0.05 (−0.32, 0.21)
I was arrested or convicted of a crime, or I spent time in jail or prison (yes vs. no)	0.03 (−1.18, 1.25)	0.24 (−1.01, 1.49)	−0.88 (−2.19, 0.44)	−0.77 (−2.07, 0.54)	0.19 (−0.85, 1.24)	0.40 (−0.75, 1.54)
I gave up on politics (yes vs. no)	0.10 (−0.23, 0.42)	0.26 (−0.02, 0.53)	0.01 (−0.46, 0.47)	0.26 (−0.13, 0.64)	0.02 (−0.39, 0.43)	0.16 (−0.20, 0.52)
My political beliefs changed a lot (yes vs. no)	**−0.44 (−0.85**,** −0.02)**^**‡**^	**−0.53 (−0.97**,** −0.10)**	−0.68 (−1.38, 0.02)	**−0.74 (−1.39**,** −0.08)**	−0.38 (−0.84, 0.07)	−0.44 (−0.94, 0.06)
I decided there was too much violence in my life (yes vs. no)	−0.09 (−0.63, 0.44)	−0.09 (−0.62, 0.45)	−0.20 (−0.85, 0.46)	−0.11 (−0.70, 0.47)	−0.04 (−0.73, 0.64)	−0.01 (−0.69, 0.68)
I had a positive experience with someone I thought was my enemy (yes vs. no)	0.02 (−0.35, 0.38)	0.05 (−0.28, 0.39)	0.10 (−0.40, 0.60)	0.26 (−0.12, 0.65)	0.11 (−0.36, 0.59)	0.20 (−0.24, 0.64)
**Number of life events**						
0	**Reference**	N/A	**Reference**	N/A	**Reference**	N/A
1	0.01 (−0.36, 0.39)	N/A	0.30 (−0.15, 0.74)	N/A	0.05 (−0.44, 0.54)	N/A
2	−0.05 (−0.41, 0.3)	N/A	0.02 (−0.38, 0.42)	N/A	−0.02 (−0.46, 0.42)	N/A
3	−0.02 (−0.42, 0.37)	N/A	0.03 (−0.36, 0.43)	N/A	−0.01 (−0.54, 0.52)	N/A
4	−0.41 (−0.83, 0.02)	N/A	−0.15 (−0.66, 0.36)	N/A	**−0.54 (−1.06**,** −0.03)**	N/A
≥ 5	0.12 (−0.29, 0.53)	N/A	0.29 (−0.18, 0.77)	N/A	0.21 (−0.30, 0.72)	N/A
**Event cluster** ^§^						
**Cluster 1** (yes vs. no)	0.06 (−0.15, 0.26)	0.07 (−0.14, 0.28)^¶^	0.21 (−0.05, 0.48)	0.20 (−0.06, 0.47)	0.10 (−0.17, 0.37)	0.13 (−0.16, 0.41)
**Cluster 2** (yes vs. no)	0.01 (−0.22, 0.23)	0.00 (−0.22, 0.22)	−0.07 (−0.37, 0.22)	−0.08 (−0.37, 0.21)	−0.05 (−0.36, 0.26)	−0.07 (−0.38, 0.24)
**Cluster 3** (yes vs. no)	−0.03 (−0.24, 0.18)	−0.04 (−0.26, 0.17)	0.09 (−0.18, 0.35)	0.05 (−0.21, 0.32)	−0.03 (−0.31, 0.25)	−0.05 (−0.35, 0.24)

Information on the relationship between change scores and change in responses to political violence questions is given in Table S2 (see Additional File 1, Supplement). For reference, an adjusted mean difference in change scores of 0.26 (the largest positive difference in this table) corresponds to a mean change of 0.07 points per question. A mean difference in change scores of −0.53 (the largest negative difference in this table) corresponds to a mean change of −0.31 points per question

^*^ Results are from a linear regression model which accounts for complex survey weights. Each life event is included in a separate model. Models are adjusted for age, gender, race and ethnicity, education, income, region, marital status, work status, having children in the household, political ideology, firearm ownership, and veteran status

^†^ Additionally adjusted for all other life events

^‡^ Statistically significant change scores (*P* < 0.05) are in bold font

^§^ Cluster 1 events were both positive and negative and concerned the partner/marriage relationship, family and extended family, employment, and health status. Cluster 2 events were negative and concerned financial status, crime and violence, mental health, and politics. Cluster 3 events were positive and concerned financial status, education, relationships with others, and social networks

^¶^ Additionally adjusted for all other clusters

Among respondents who reported in Wave 1 that they strongly or very strongly approved of at least 1 extremist organization or movement (Table 5; Fig. 2), “my political beliefs changed a lot” was associated with a large decrease in support for political violence in Model 2 (aMD − 0.53, 95% CI −0.97, −0.10) that remained statistically significant for approvers of left-wing, but not right-wing, organizations or movements.

Across all 3 subgroups of respondents, findings for the justification, willingness, and firearm subsets of the political violence measures (see Additional File 1, Supplement, Tables S10-S12) were similar to those for all respondents (see Fig. 1 and Additional File 1, Supplement, Table S9).

## Discussion

Contrary to our hypotheses, there was only infrequent association between the occurrence of the life events studied here and change in support for political violence. Most of the associations we observed in unadjusted analyses were with increases in support, and these were attenuated or no longer statistically significant in adjusted models. We observed no associations with individual events among respondents who in Wave 1 considered violence to be usually or always justified to advance at least 1 political objective, a concerning subgroup with a high level of support for political violence [12]. A change in political beliefs was associated with decreased support for political violence among those who supported left-wing, but not right-wing, violent extremist groups. Experiencing a large number of events was associated with increased support, likely because those who reported doing so were also particularly likely to report experiencing events that had large individual effect sizes.

A few clear signals emerge from comparison of findings across all analyses for the study cohort as a whole. An improved financial situation and a substantial change in political beliefs are associated with decreased support for political violence. Starting or completing an educational activity, arrest and incarceration, and experiencing a large number of these events are associated with increased support. The same is true of “I gave up on politics,” from which we infer that support for political violence increases when achievement of political objectives through the political process appears futile. There are important gender differences, such as with an improved financial situation (a decrease for men but not women) and educational activity (an increase for women but not men). In other cases, such as a decrease associated with having a child or grandchild and an increase associated with under- or unemployment, there are persistent associations that do not reach statistical significance.

Where we did observe associations between life events and support for political violence, our findings are largely concordant with those of earlier studies of disinvolvement with extremist or terrorist violence [14–20]. What might account for the discordances? One potential explanation is methodological. The terrorism literature largely comprises small, uncontrolled qualitative studies, and it is possible that some life events reported by participants in those studies who had become disinvolved with violence occurred just as frequently among nonparticipants who remained involved.

Alternatively, associations seen in the earlier studies of participants in political violence might be lost in this study of the general population; even in our high-risk subgroups, it is unlikely that more than a small minority have participated in political violence. Demographic differences in study populations are another potential source of difference. The median age of our respondents was above 55 years, and participants in the terrorist literature were often in their 20 s and 30s. This raises the possibility of something akin to survivor bias in our population, particularly among those who supported political violence in 2022. Individuals whose support was susceptible to influence by life events might have experienced those events in prior years—many events were common among our respondents—and might already have undergone the change we were investigating by the time of our survey in 2022. There are cultural differences as well; much of the terrorism literature involves non-US populations.

Some findings were unexpected and require further study, perhaps best through interviews. The increase in support for political violence among women (but not men) who started or completed an educational activity or “had a positive experience with someone I thought was my enemy” is a good example. We also note here the repeated but statistically non-significant association between marriage or engagement and increased support for political violence among women, but not men, and the association among men between making “some good new friends” and increased willingness to engage in political violence. These findings suggest radicalization mediated by close personal relationships or, more broadly, the bonding form of social capital [38]. That interpretation is reinforced by a separate study of this cohort, in which membership in a large social network with uniform beliefs was associated with increased support for political violence [39]. Others have also found that higher levels of some forms of social capital are associated with higher levels of support for political violence [40, 41].

A few specific findings offer immediate guidance for prevention efforts. Policies that are frequently implemented at the population level, such as those that improve employment, income, and access to services, might reduce risk for political violence. The same might be true for those interventions and others implemented with high-risk individuals, as hospital-based violence intervention programs have demonstrated for other forms of violence [42–44]. Preventing political violence could be added to the objectives of efforts to increase engagement with nonviolent political activity and to reduce incarceration or change its conditions. Increased efforts to change the political beliefs of those who support extremist organizations and movements are also justified by our results, as is further research to identify characteristics associated with openness to change among such individuals [14–19, 38].

The potential yield of individual change for reducing risk of political violence is supported by related findings from 2024’s Wave 3 of this survey. Among respondents who considered it very or extremely likely that they would participate as a combatant should civil war break out in the United States, 44.5% said that their expectation would convert to not likely if this were urged by their family, and 20%−30% said that urging by friends, respected community or religious leaders, or the media would have this effect [45].

That said, the relative lack of association seen here between life events and change in support for political violence is reinforced by the repeated finding that violence, including political violence in this cohort, is strongly associated with characteristics such as racism, sexism, homonegativity, transphobia, xenophobia, Islamophobia, and antisemitism [46–53]. Such characteristics are resistant to change, and intervening directly on them has little effect [1]. Except where clear alternatives are identified, prevention efforts may need to focus on uncoupling such durable characteristics in high-risk individuals from their associated violent behaviors. Characteristics that constitute barriers to change have also been identified and will need to be included in intervention planning [54, 55].

### Limitations

Perhaps the most important limitation to this analysis is our inability to answer the question, What magnitude of change in support for political violence associated with the experience of life events is big enough to matter? There is no reference standard. The authors suggest that change scores of ± 0.6 might be useful threshold values across the 35 political violence questions. These scores correspond to a full-point change toward increased support on roughly 1 question in 4 or toward decreased support on roughly 1 question in 3. That 1-in-4 threshold would be associated with different change scores for the question subsets. This remains a topic for further consideration and research.

Several technical limitations exist as well. The findings are subject to sampling error and nonresponse bias. Arguably, nonresponse was most important in Wave 1; the 84% response rate for Wave 2 was high. A few of our life events and political violence measures were uncommon experiences, and limited statistical power may be the cause of the many associations that do not reach statistical significance. We relied on imputation for political violence measures when computing change scores. This reduced precision in those estimates, but missingness was uncommon.

External events (or their absence) may have affected our findings. In 2022, widely publicized mass shootings occurred in Buffalo, NY and Uvalde, TX while the survey was in the field; there were no comparable events during the fielding of the 2023 survey. The Buffalo shooting is understood to have been a race-related hate crime motivated by great replacement thinking and may have affected respondents’ views on race and violence. In 2023, the survey closed just before the federal criminal indictment of Donald Trump was handed down; support for violence to return him to the White House increased immediately thereafter [56]. In both years, Russia’s war against Ukraine may have influenced responses on violence and democracy.

## Conclusions

Findings from this large, nationally representative cohort survey indicate that some life events, and the experience of large numbers of such events, are associated with important changes in support for political violence in the United States. The findings support and can guide efforts to increase the frequency of events associated with a decrease in support for violence and decrease the frequency or alter the effects of events associated with an increase. Further research to clarify ambiguous or unexpected findings is needed.

## Supplementary Information

Supplementary Material 1

## Data Availability

The datasets generated and/or analyzed during the current study are not publicly available as analyses are continuing but will be made available to qualified researchers subject to the terms of a data use agreement.

## Abbreviations

SD: Standard deviation
CI: Confidence interval
aMD: Adjusted mean difference
