# Supplementary material for: Life events and change in support for political violence in the United States: findings from a 2023 nationally representative survey
Source: Inj Epidemiol. 2025 Dec 27;13:9. doi: 10.1186/s40621-025-00652-3 (PMC12853587; doi:10.1186/s40621-025-00652-3)
Supplement: Supplementary file 1 — Supplementary Material 1 [file 40621_2025_652_MOESM1_ESM.pdf]

Supplement

Life Events and Change in Support for Political Violence in the United States:

Findings from a 2023 Nationally Representative Survey

This supplement has been provided by the authors to give readers additional information about the work.

Page	Title
2	Questions that supplied data for this study
10	References
11	Table S1. Loadings for principal components for the primary analysis, 2023
13	Table S2. Relationship between change scores and change in responses to political violence questions
15	Table S3. Extremist organizations and movements
16	Table S4. Event cluster matrix structure
17	Table S5. Event cluster summary for 3 clusters
18	Table S6. Sociodemographic characteristics (weighted) of respondents
20	Table S7. Sociodemographic characteristics (unweighted) of respondents and non-respondents in the 2022 and 2023 surveys
22	Table S8. Mean scores and mean change scores across categories of covariates
25	Table S9. Experience of life events and adjusted mean differences in change scores for political violence question subsets
27	Table S10. Experience of life events and adjusted mean differences in change scores for political violence question subsets, by gender
29	Table S11. Experience of life events and adjusted mean differences in change scores for political violence question subsets, among respondents who indicated violence was usually or always justified to advance at least 1 political objective in 2022
31	Table S12. Experience of life events and adjusted mean differences in change scores for political violence question subsets, among respondents who strongly or very strongly approved of at least one extremist organization/movement in 2022
33	Figure S1. Scree plots for the first factor of the PCA in 2023 for all 35 political violence questions combined and for the justification, willingness, and firearm subsets
35	Figure S2. Distribution of change scores for the main analysis
36	Figure S3. Cluster summaries and inter-cluster correlations for all cluster solutions

QUESTIONS THAT SUPPLIED DATA FOR THIS STUDY

Response options are presented here in order from negative to positive (e.g., “not important” to “extremely important”). Respondents were randomized 1:1 to receive responses in that order or the reverse.

In the list below, questions or items that were repeated or adapted from prior surveys by other investigators contain citations to those surveys.

Domain 1: Life events

Q: Thinking back over the year since you responded to our survey last May or June, have any of these things happened in your life? Please indicate yes or no for each one.

a. I had a child or grandchild.

b. I started a new romantic relationship, or an existing relationship grew stronger.

c. I got married or engaged.

d. I got a new job, or a better job.

e. I lost my job or had my hours cut back.

f. I suffered a serious illness.

g. My partner, a close family member, or a close friend suffered a serious illness or died.

1. No
2. Yes

Q: Again, thinking back over the past year, have any of these things happened in your life? Please indicate yes or no for each one.

h. Things improved for me financially.

i. Things got worse for me financially.

j. I started a new educational activity or completed one.

k. I started a new social or community service activity.

l. I made some good new friends.

1. No

2. Yes

Q: And again, have any of these things happened in your life over the past year? Please indicate yes or no for each one.

m. I got burned out.

n. I was arrested or convicted of a crime, or I spent time in jail or prison.

o. I gave up on politics.

p. My political beliefs changed a lot.

q. I decided there was too much violence in my life.

r. I had a positive experience with someone I thought was my enemy.

1. No
2. Yes

Domain 2: democracy in the United States

Now we'd like to ask you a few questions about the United States as you see it now, in 2023.

Q: When thinking about democracy in the United States these days, do you believe…?[1]

1. There is a serious threat to our democracy.
2. There may be a threat to our democracy, but it is not serious.
3. There is no threat to our democracy.

Q: How important do you think it is for the United States to remain a democracy?[2]

1. Not important
2. Somewhat important
3. Very important
4. Extremely important

Q: How much do you agree or disagree with the following statements about democracy in the United States?

a. Democracy is the best form of government.[3]

b. These days, American democracy only serves the interests of the wealthy and powerful.[4]

c. Having a strong leader for America is more important than having a democracy.

d. The 2020 election was stolen from Donald Trump, and Joe Biden is an illegitimate president.

1. Do not agree
2. Somewhat agree
3. Strongly agree
4. Very strongly agree

Q: People have different views about violence in the United States. How much do you agree or disagree with each of the following statements?

a. If elected leaders will not protect American democracy, the people must do it themselves, even if it requires taking violent actions.[4]

b. Because things have gotten so far off track, true American patriots may have to resort to violence in order to save our country.[5]

c. Our American way of life is disappearing so fast that we may have to use force to save it.[4]

1. Do not agree
2. Somewhat agree
3. Strongly agree
4. Very strongly agree

Q: How much do you agree or disagree with each of the following statements?

a. In the next few years, there will be civil war in the United States.[6]

1. Do not agree
2. Somewhat agree

3. Strongly agree

4. Very strongly agree

Domain 3: American society and institutions

The next few questions are about your views of American society.

Q: How much do you agree or disagree with each of the following statements about people in the United States today?

a. White people benefit from advantages in society that Black people do not have.[4]

b. Discrimination against whites is as big a problem as discrimination against Blacks and other minorities.[4]

c. A group of people in this country is trying to replace native-born Americans with immigrants and people of color who share their political views. (In 2022: In America, native-born white people are being replaced by immigrants.)

d. Having more Black Americans, Latinos, and Asian Americans is good for the country.[7]

1. Do not agree

2. Somewhat agree

3. Strongly agree

4. Very strongly agree

Q: People have many different views about society in the United States. How much do you agree or disagree with each of the following?

a. The government, media, and financial worlds in the U.S. are controlled by a group of Satan-worshipping pedophiles who run a global child sex trafficking operation.[5]

b. There is a storm coming soon that will sweep away the elites in power and restore the rightful leaders.[5]

c. The chaos in America today is evidence that we are living in what the Bible calls “the end times.”[8]

1. Do not agree

2. Somewhat agree

3. Strongly agree

4. Very strongly agree

Domain 4: political violence

Now we have a few questions about the use of force or violence. "Force or violence" means physical force strong enough that it could cause pain or injury to a person. A reminder: your responses will be kept confidential and anonymous.

Q: People sometimes talk about using force or violence to achieve political objectives. In general, what do you think about using force or violence to advance an important political objective that you support—is it…?

1. Never justified

2. Sometimes justified

3. Usually justified

4. Always justified

Q: Your view of the use of force or violence to advance an important political objective might depend on the specific objective that was involved. What do you think about the use of force or violence in the following situations—is it never justified, sometimes justified, usually justified, or always justified?

a. To return Donald Trump to the presidency this year

b. To stop an election from being stolen

c. To stop people who do not share my beliefs from voting

d. To prevent discrimination based on race or ethnicity

e. To preserve an American way of life based on Western European traditions

f. To oppose the government when it does not share my beliefs

g. To oppose the government when it tries to take private land for public purposes

1. Never justified

2. Sometimes justified
3. Usually justified
4. Always justified

Q: You said that in general, the use of force or violence was [response inserted] to advance an important political objective that you support. Your opinion might depend on the specific objective that was involved. What do you think about the use of force or violence in the following situations—is it never justified, sometimes justified, usually justified, or always justified?

[Items 1a-4b were paired in 2022, and each respondent was presented with 1 item from each pair. In 2023 all items were presented to all respondents.]

1a. To stop voter fraud

1b. To stop voter intimidation

2a. To stop police violence

2b. To reinforce the police

3a. To stop illegal immigration

3b. To keep our borders open

4a. To stop a protest or demonstration

4b. To support a protest or demonstration

5. To preserve the American way of life I believe in

6. To oppose Americans who do not share my beliefs

1. Never justified
2. Sometimes justified
3. Usually justified

4. Always justified

The next questions are about your personal willingness to use force or violence.

(Questions asked of respondents who endorsed at least 1 use of violence to achieve a specific political objective.)

Q: In a situation where you think force or violence is justified to advance an important political objective, how willing would you personally be to use force or violence in each of these ways?

a. To damage property

b. To threaten or intimidate a person

c. To injure a person

d. To kill a person

1. Not willing
2. Somewhat willing
3. Very willing
4. Completely willing

Q: In a situation where you think force or violence is justified to advance an important political objective, how willing would you personally be to use force or violence against a person because they are…

a. An elected federal or state government official

b. An elected local government official

c. A public health official

d. A member of the military or National Guard

e. A police officer

f. A person who does not share your race or ethnicity

g. A person who does not share your religion

h. An election worker, such as a poll worker or vote counter

i. A person who does not share your political beliefs

1. Not willing
2. Somewhat willing
3. Very willing
4. Completely willing

(Question asked of all respondents.)

Q: Thinking now about the future and all the changes it might bring, how likely is it that you will use a gun in any of the following ways in the next few years—in a situation where you think force or violence is justified to advance an important political objective?

a. I will be armed with a gun.

b. I will carry a gun openly, so that people know I am armed.

c. I will threaten someone with a gun.

d. I will shoot someone with a gun.

1. Not likely
2. Somewhat likely
3. Very likely
4. Extremely likely

REFERENCES

1. NPR/PBS NewsHour/Marist National Poll. Trust in elections, threat to democracy, November 2021. 2021 November 1. https://maristpoll.marist.edu/polls/npr-pbs-newshour-marist-national-poll-trust-in-elections-threat-to-democracy-biden-approval-november-2021/.

2. Grinnell College National Poll. 52% of Americans believe democracy facing “major threat.” Study #2243. 2021 October 20. https://www.grinnell.edu/news/52-americans-believe-democracy-facing-major-threat.

3. The Economist/YouGov Poll. 2021 June 13-16. https://docs.cdn.yougov.com/uagnfc262c/econTabReport.pdf.

4. Survey Center on American Life. January 2021 American Perspectives Survey topline questionnaire. https://www.americansurveycenter.org/wp-content/uploads/2021/03/January-2021-APS-Topline-Questionnaire.pdf.

5. Public Religion Research Institute. The persistence of Q-Anon in the post-Trump era: an analysis of who believes the conspiracies. 2022 Feb 24. https://www.prri.org/research/the-persistence-of-qanon-in-the-post-trump-era-an-analysis-of-who-believes-the-conspiracies/.

6. Zogby. Will the US have another civil war? 2021 Feb 4. https://zogbyanalytics.com/news/997-the-zogby-poll-will-the-us-have-another-civil-war

7. Pew Research Center. Americans see advantages and challenges in country’s growing racial and ethnic diversity. 2019 May. https://www.pewresearch.org/social-trends/2019/05/08/americans-see-advantages-and-challenges-in-countrys-growing-racial-and-ethnic-diversity/.

8. IFYC – PRRI Survey on Religion & COVID-19 Vaccine Trust. 2021 March. https://www.prri.org/wp-content/uploads/2021/05/Topline-IFYC-PRRI-Survey-on-Religion-and-COVID-19-Vaccine-Trust-v2_final.pdf.

Table S1. Loadings for principal components for the primary analysis, 2023

Subset*	Political violence item	PC1	PC2	PC3	PC4	PC5	PC6
J	In general...to advance an important political objective that you support	0.64	0.08	-0.14	0.45	-0.18	-0.08
J	To stop voter fraud	0.80	0.17	0.43	-0.15	0.01	0.18
J	To stop voter intimidation	0.77	-0.01	0.30	0.01	0.03	0.36
J	To stop police violence	0.74	-0.23	0.13	0.13	0.13	0.38
J	To reinforce the police	0.75	-0.04	0.12	-0.48	-0.02	-0.01
J	To stop illegal immigration	0.76	0.09	0.20	-0.47	-0.04	-0.01
J	To keep our borders open	0.67	-0.06	0.15	-0.01	0.44	-0.02
J	To stop a protest or demonstration	0.75	0.04	0.14	-0.23	0.28	-0.23
J	To support a protest or demonstration	0.77	0.05	0.29	0.19	0.30	0.12
J	To preserve the American way of life I believe in	0.78	0.14	0.23	-0.25	-0.09	-0.07
J	To oppose Americans who do not share my beliefs	0.82	0.21	0.20	0.20	0.23	-0.12
J	To return Donald Trump to the presidency this year	0.74	0.31	0.25	0.03	-0.12	-0.12
J	To stop an election from being stolen	0.78	0.19	0.26	0.04	-0.32	0.08
J	To stop people who do not share my beliefs from voting	0.80	0.24	0.11	0.29	0.11	-0.27
J	To prevent discrimination based on race or ethnicity	0.71	-0.07	0.15	0.42	-0.13	0.21
J	To preserve an American way of life based on Western European traditions	0.75	0.23	0.15	-0.05	-0.29	-0.26
J	To oppose the government when it does not share my beliefs	0.79	0.20	0.14	0.26	-0.15	-0.11
J	To oppose the government when it tries to take private land for public purposes	0.75	0.09	0.05	0.07	-0.38	0.02
W	To damage property	0.88	-0.30	-0.26	0.00	-0.05	0.05
W	To threaten or intimidate a person	0.90	-0.25	-0.26	-0.04	-0.04	0.01
W	To injure a person	0.91	-0.24	-0.25	-0.03	-0.04	0.01
W	To kill a person	0.93	-0.22	-0.21	-0.06	-0.01	-0.02
W	An elected federal or state government official	0.96	-0.20	-0.11	-0.02	-0.03	-0.02
W	An elected local government official	0.96	-0.20	-0.11	-0.02	-0.03	-0.02
W	A public health official	0.97	-0.16	-0.07	-0.04	-0.01	-0.05
W	A member of the military or National Guard	0.95	-0.22	-0.13	-0.03	0.01	-0.01
W	A police officer	0.93	-0.25	-0.18	0.02	0.02	0.03
W	A person who does not share your race or ethnicity	0.97	-0.17	-0.07	-0.02	0.02	-0.05
W	A person who does not share your religion	0.97	-0.17	-0.05	-0.02	0.01	-0.06
W	An election worker, such as a poll worker or vote counter	0.97	-0.18	-0.05	-0.03	0.02	-0.04

Table S1, continued.

**Subset***	**Political violence item**	**PC1**	**PC2**	**PC3**	**PC4**	**PC5**	**PC6**
W	A person who does not share your political beliefs	0.97	-0.20	-0.09	0.00	0.00	-0.03
F	I will be armed with a gun	0.44	0.63	-0.40	-0.22	-0.13	0.24
F	I will carry a gun openly, so that people know	0.52	0.66	-0.30	-0.12	0.03	0.16
F	I will threaten someone with a gun	0.71	0.49	-0.29	0.13	0.24	-0.05
F	I will shoot someone with a gun	0.63	0.53	-0.32	0.06	0.21	0.04

* J denotes justification subset; W denotes willingness subset; F denotes firearm subset

Table S2. Relationship between change scores and change in responses to political violence questions, for all questions and question subsets

Range of change score	All questions (n = 35)				Justified questions (n = 18)			
	N	Mean (SD) of change score	Mean (SD) of summed change in responses (points)	Mean (SD) of change in responses per question (points)	N	Mean (SD) of change score	Mean (SD) of summed change in responses (points)	Mean (SD) of change in responses per question (points)
<-1	903	-1.33 (0.56)	-20.77 (8.46)	-0.59 (0.24)	473	-1.59 (0.66)	-13.70 (5.24)	-0.76 (0.29)
≥-1 to -0.9	648	-0.94 (0.03)	-14.56 (1.37)	-0.42 (0.04)	112	-0.95 (0.03)	-8.45 (1.70)	-0.47 (0.09)
≥-0.9 to -0.8	106	-0.87 (0.03)	-13.30 (2.93)	-0.38 (0.08)	122	-0.85 (0.03)	-7.96 (1.16)	-0.44 (0.06)
≥-0.8 to -0.7	54	-0.75 (0.03)	-13.80 (2.37)	-0.39 (0.07)	168	-0.75 (0.03)	-7.04 (1.48)	-0.39 (0.08)
≥-0.7 to -0.6	79	-0.65 (0.03)	-11.97 (2.48)	-0.34 (0.07)	221	-0.65 (0.03)	-6.28 (1.34)	-0.35 (0.07)
≥-0.6 to -0.5	99	-0.55 (0.03)	-10.90 (2.22)	-0.31 (0.06)	301	-0.55 (0.03)	-5.21 (1.11)	-0.29 (0.06)
≥-0.5 to -0.4	156	-0.45 (0.03)	-9.30 (1.72)	-0.27 (0.05)	391	-0.45 (0.03)	-4.32 (1.23)	-0.24 (0.07)
≥-0.4 to -0.3	197	-0.34 (0.03)	-7.44 (1.66)	-0.21 (0.05)	549	-0.35 (0.03)	-3.44 (1.09)	-0.19 (0.06)
≥-0.3 to -0.2	366	-0.25 (0.03)	-5.86 (1.49)	-0.17 (0.04)	752	-0.24 (0.02)	-2.51 (1.06)	-0.14 (0.06)
≥-0.2 to -0.1	624	-0.15 (0.03)	-3.98 (1.50)	-0.11 (0.04)	936	-0.14 (0.02)	-1.54 (1.01)	-0.09 (0.06)
≥-0.1 to 0.0	896	-0.05 (0.03)	-2.21 (1.31)	-0.06 (0.04)	1618	-0.03 (0.02)	-0.38 (0.82)	-0.02 (0.05)
≥0.0 to 0.1	1115	0.05 (0.03)	-0.51 (1.27)	-0.01 (0.04)	754	0.05 (0.03)	-0.11 (1.06)	-0.01 (0.06)
≥0.1 to 0.2	1636	0.13 (0.03)	0.54 (1.20)	0.02 (0.03)	572	0.15 (0.03)	0.40 (1.35)	0.02 (0.07)
≥0.2 to 0.3	452	0.25 (0.03)	2.43 (1.68)	0.07 (0.05)	465	0.25 (0.03)	1.04 (1.42)	0.06 (0.08)
≥0.3 to 0.4	311	0.35 (0.03)	3.78 (2.00)	0.11 (0.06)	353	0.35 (0.03)	1.54 (1.64)	0.09 (0.09)
≥0.4 to 0.5	198	0.45 (0.03)	5.36 (1.94)	0.15 (0.06)	263	0.45 (0.03)	2.23 (1.59)	0.12 (0.09)
≥0.5 to 0.6	133	0.55 (0.03)	6.92 (1.68)	0.20 (0.05)	211	0.55 (0.03)	2.81 (1.70)	0.16 (0.09)
≥0.6 to 0.7	110	0.65 (0.03)	8.02 (2.09)	0.23 (0.06)	171	0.65 (0.03)	3.49 (1.66)	0.19 (0.09)
≥0.7 to 0.8	80	0.75 (0.03)	9.28 (2.33)	0.27 (0.07)	112	0.75 (0.03)	4.09 (1.55)	0.23 (0.09)
≥0.8 to 0.9	75	0.85 (0.03)	10.36 (2.83)	0.30 (0.08)	107	0.84 (0.03)	4.40 (1.98)	0.24 (0.11)
≥0.9 to 1.0	74	0.95 (0.03)	11.99 (2.59)	0.34 (0.07)	86	0.96 (0.03)	5.28 (2.11)	0.29 (0.12)
≥1.0	1073	1.39 (0.66)	18.53 (9.77)	0.53 (0.28)	648	1.80 (0.80)	10.67 (5.92)	0.59 (0.33)

Table S2, continued.

	Willingness questions (n = 13)				Firearm questions (n = 4)			
**Range of change score**	**N**	**Mean (SD) of change score**	**Mean (SD) of summed change in responses (points)**	**Mean (SD) of change in responses per question (points)**	**N**	**Mean (SD) of change score**	**Mean (SD) of summed change in responses (points)**	**Mean (SD) of change in responses per question (points)**
<-1	1650	-1.71 (0.44)	-13.40 (3.13)	-1.03 (0.24)	457	-2.07 (1.54)	-2.85 (1.88)	-0.71 (0.47)
≥-1 to -0.9	20	-0.96 (0.02)	-6.95 (0.89)	-0.53 (0.07)	19	-0.94 (0.04)	-1.05 (1.03)	-0.26 (0.26)
≥-0.9 to -0.8	18	-0.85 (0.03)	-6.67 (0.49)	-0.51 (0.04)	37	-0.84 (0.02)	-1.65 (0.63)	-0.41 (0.16)
≥-0.8 to -0.7	40	-0.75 (0.03)	-5.73 (0.64)	-0.44 (0.05)	94	-0.76 (0.04)	-1.24 (0.83)	-0.31 (0.21)
≥-0.7 to -0.6	32	-0.64 (0.03)	-4.88 (0.42)	-0.38 (0.03)	66	-0.65 (0.01)	-0.92 (0.37)	-0.23 (0.09)
≥-0.6 to -0.5	31	-0.55 (0.03)	-4.03 (1.52)	-0.31 (0.12)	46	-0.54 (0.02)	-0.67 (0.52)	-0.17 (0.13)
≥-0.5 to -0.4	63	-0.45 (0.03)	-3.78 (0.49)	-0.29 (0.04)	303	-0.44 (0.02)	-0.94 (0.32)	-0.24 (0.08)
≥-0.4 to -0.3	97	-0.34 (0.03)	-2.78 (0.68)	-0.21 (0.05)	42	-0.35 (0.03)	-0.17 (0.58)	-0.04 (0.15)
≥-0.3 to -0.2	77	-0.24 (0.03)	-2.22 (0.55)	-0.17 (0.04)	112	-0.23 (0.01)	0.02 (0.23)	0.00 (0.06)
≥-0.2 to -0.1	167	-0.17 (0.02)	-1.87 (0.34)	-0.14 (0.03)	169	-0.14 (0.02)	0.03 (0.23)	0.01 (0.06)
≥-0.1 to 0.0	416	-0.05 (0.02)	-0.94 (0.34)	-0.07 (0.03)	6140	-0.07 (0.00)	0.00 (0.05)	0.00 (0.01)
≥0.0 to 0.1	3776	0.09 (0.01)	0.00 (0.07)	0.00 (0.01)	32	0.06 (0.02)	0.66 (0.60)	0.16 (0.15)
≥0.1 to 0.2	72	0.17 (0.03)	0.89 (0.43)	0.07 (0.03)	82	0.16 (0.02)	0.98 (0.22)	0.24 (0.06)
≥0.2 to 0.3	1402	0.24 (0.02)	0.29 (0.49)	0.02 (0.04)	521	0.24 (0.01)	1.01 (0.19)	0.25 (0.05)
≥0.3 to 0.4	199	0.34 (0.02)	2.02 (0.31)	0.16 (0.02)	58	0.35 (0.02)	1.14 (0.61)	0.28 (0.15)
≥0.4 to 0.5	120	0.45 (0.02)	2.97 (0.33)	0.23 (0.03)	117	0.43 (0.03)	1.33 (0.49)	0.33 (0.12)
≥0.5 to 0.6	57	0.56 (0.03)	3.95 (0.40)	0.30 (0.03)	143	0.55 (0.01)	1.93 (0.37)	0.48 (0.09)
≥0.6 to 0.7	33	0.64 (0.03)	4.36 (0.78)	0.34 (0.06)	43	0.66 (0.02)	1.86 (0.52)	0.47 (0.13)
≥0.7 to 0.8	50	0.74 (0.03)	5.34 (0.56)	0.41 (0.04)	237	0.72 (0.01)	1.91 (0.42)	0.48 (0.10)
≥0.8 to 0.9	20	0.84 (0.03)	6.05 (0.60)	0.47 (0.05)	130	0.86 (0.01)	2.77 (0.59)	0.69 (0.15)
≥0.9 to 1.0	25	0.96 (0.03)	7.00 (0.41)	0.54 (0.03)	33	0.96 (0.03)	2.24 (0.87)	0.56 (0.22)
≥1.0	1020	2.07 (0.59)	13.90 (4.53)	1.07 (0.35)	504	2.23 (1.42)	4.32 (2.13)	1.08 (0.53)

Table S3. Extremist organizations and movements

Organizations
**Right wing**
Proud Boys
Oath Keepers
Three Percenters
QAnon
**Left wing**
Redneck Revolt
**Movements**
**Right wing**
White supremacy movement
Christian nationalist movement
Militia movement
Boogaloo movement
**Left wing**
Antifascist (Antifa) movement
Anarchist movement

Table S4. Event cluster* structure matrix

Life event	Correlation between Variable and Cluster		
	Cluster 1	Cluster 2	Cluster 3
I had a child or grandchild.	0.47	0.07	0.07
I started a new romantic relationship, or an existing relationship grew stronger.	0.56	0.14	0.19
I got married or engaged.	0.68	0.13	0.15
I got a new job, or a better job.	0.54	0.13	0.22
I lost my job or had my hours cut back.	0.63	0.23	0.10
I suffered a serious illness.	0.51	0.15	0.08
My partner, a close family member, or a close friend suffered a serious illness or died.	0.39	0.13	0.09
Things improved for me financially.	0.13	-0.04	0.45
Things got worse for me financially.	0.13	0.48	-0.03
I started a new educational activity or completed one.	0.15	0.13	0.54
I started a new social or community service activity.	0.13	0.11	0.63
I made some good new friends.	0.14	0.10	0.69
I got burned out.	0.17	0.54	0.13
I was arrested or convicted of a crime, or I spent time in jail or prison.	0.22	0.48	0.16
I gave up on politics.	0.09	0.58	0.08
My political beliefs changed a lot.	0.10	0.54	0.10
I decided there was too much violence in my life.	0.13	0.57	0.13
I had a positive experience with someone I thought was my enemy.	0.13	0.21	0.50

* Cluster 1 events were both positive and negative and concerned the partner/marriage relationship, family and extended family, employment, and health status. Cluster 2 events were negative and concerned financial status, crime and violence, mental health, and politics. Cluster 3 events were positive and concerned financial status, education, relationships with others, and social networks.

Table S5. Event cluster summary* for 3 clusters

Event Cluster	Members	Cluster Variation	Variation Explained	Proportion Explained	Second Eigenvalue	Intra-cluster correlations	
						**2**	**3**
1	7	7	2.10	0.30	1.01	0.26	0.24
2	6	6	1.70	0.28	1.02	--	0.18
3	5	5	1.62	0.32	0.94	--	--
Total variation explained = 5.41 Proportion = 0.30							

* Each summary explains the variation in each group of variables, the number of variables in each cluster, and the proportion of variance explained by each cluster component, with larger values indicating a better fit for the cluster variables. Cluster 1 events were both positive and negative and concerned the partner/marriage relationship, family and extended family, employment, and health status. Cluster 2 events were negative and concerned financial status, crime and violence, mental health, and politics. Cluster 3 events were positive and concerned financial status, education, relationships with others, and social networks.

Table S6. Sociodemographic characteristics (weighted) of respondents

Characteristic	2023 Respondents* (n= 9385)	
	Unweighted n	Weighted % (95% CI)
**Age**		
18-24	310	10.3 (9.2, 11.5)
25-34	856	16.8 (15.6, 18.0)
35-44	1252	18.5 (17.4, 19.6)
45-54	1255	14.3 (13.4, 15.2)
55-64	2043	17.6 (16.7, 18.5)
65-74	2342	14.5 (13.8, 15.3)
75+	1327	8.0 (7.4, 8.5)
Non-response	0	0.0 (0.0, 0.0)
**Gender**		
Female	3866	50.7 (49.4, 52.1)
Male	5340	47.0 (45.7, 48.4)
Transgender	45	0.5 (0.3, 0.7)
Non-binary	59	0.8 (0.5, 1.0)
Other	21	0.3 (0.1, 0.5)
Non-response	54	0.7 (0.4, 0.9)
**Race/Ethnicity**		
White, Non-Hispanic	7014	62.7 (61.2, 64.1)
Black, Non-Hispanic	748	12.0 (10.9, 13.0)
Hispanic, any race	1016	16.9 (15.7, 18.1)
American Indian or Alaska Native, Non-Hispanic	47	1.1 (0.7, 1.5)
Asian American or Pacific Islander, non-Hispanic	277	5.5 (4.7, 6.2)
Some other race, Non-Hispanic	19	0.1 (0.1, 0.2)
2+ Races, Non-Hispanic	264	1.8 (1.4, 2.2)
Non-response	0	0.0 (0.0, 0.0)
**Marital status**		
Now married	5961	56.2 (54.8, 57.6)
Widowed	582	3.9 (3.5, 4.4)
Divorced	1010	8.2 (7.6, 8.8)
Separated	122	1.4 (1.1, 1.8)
Never married	1710	30.2 (28.8, 31.6)
Non-response	0	0.0 (0.0, 0.0)
**Education**		
No high school diploma or GED	416	9.5 (8.4, 10.5)
High school graduate (diploma, GED)	2002	28.2 (26.9, 29.6)
Some college or Associate's degree	2773	27.1 (25.9, 28.3)
Bachelor's degree	2337	20.1 (19.1, 21.1)
Master’s degree or higher	1857	15.1 (14.2, 15.9)
Non-response	0	0.0 (0.0, 0.0)

Table S6, continued.

Characteristic	2023 Respondents* (n= 9385)	
	Unweighted n	Weighted % (95% CI)
**Household Income**		
Less than $10,000	233	3.9 (3.2, 4.5)
$10,000 to $24,999	727	8.9 (8.1, 9.8)
$25,000 to $49,999	1617	17.0 (15.9, 18.0)
$50,000 to $74,999	1631	16.3 (15.3, 17.4)
$75,000 to $99,999	1499	13.2 (12.3, 14.1)
$100,000 to $149,999	1734	17.9 (16.8, 18.9)
$150,000 or more	1944	22.8 (21.6, 23.9)
Non-response	0	0.0 (0.0, 0.0)
**Employment**		
Working - as a paid employee	4291	52.9 (51.6, 54.3)
Working - self-employed	709	7.2 (6.5, 8.0)
Not working - on temporary layoff from a job	35	0.5 (0.3, 0.7)
Not working - looking for work	272	5.2 (4.4, 5.9)
Not working - retired	3367	21.3 (20.4, 22.2)
Not working - disabled	286	4.5 (3.9, 5.2)
Not working - other	425	8.3 (7.4, 9.2)
Non-response	0	0.0 (0.0, 0.0)
**Census division**		
New England	374	4.7 (4.1, 5.3)
Mid-Atlantic	1001	12.6 (11.6, 13.5)
East-North Central	1370	14.3 (13.3, 15.2)
West-North Central	676	6.4 (5.8, 7.0)
South Atlantic	1881	20.5 (19.4, 21.6)
East-South Central	538	5.8 (5.1, 6.5)
West-South Central	965	11.9 (10.9, 12.8)
Mountain	825	7.6 (6.9, 8.3)
Pacific	1755	16.3 (15.3, 17.3)
Non-response	0	0.0 (0.0, 0.0)

This table first appeared as part of Table 1 in Wintemute GJ, Robinson SL, Crawford A, Tomsich EA, Reeping PM, Shev AB, Velasquez B, Tancredi D. Single-year change in views of democracy and society and support for political violence in the USA: findings from a 2023 nationally representative survey. Injury Epidemiology. 2024;11(1):20.

Table S7. Sociodemographic characteristics (unweighted) of respondents and non-respondents in the 2022 and 2023 surveys

Characteristic	2022 (Wave1)				Wave1 respondents who left the panel prior to wave2 (n = 1,807)		2023 (Wave2)			
	Respondents (n = 12,947)		Non-respondents (n = 8,318)				Respondents (n = 9,385)		Non-respondents (n = 1,755)	
	Unweighted n	Unweighted %	Unweighted n	Unweighted %	Unweighted n	Unweighted %	Unweighted n	Unweighted %	Unweighted n	Unweighted %
Age										
18-24	488	3.8	1059	12.7	86	4.8	310	3.3	92	5.2
25-34	1309	10.1	1411	17.0	210	11.6	856	9.1	243	13.8
35-44	1884	14.6	1732	20.8	326	18	1252	13.3	306	17.4
45-54	1847	14.3	1599	19.2	335	18.5	1255	13.4	257	14.6
55-64	2794	21.6	1254	15.1	391	21.6	2043	21.8	360	20.5
65-74	2952	22.8	861	10.4	313	17.3	2342	25.0	297	16.9
75+	1673	12.9	402	4.8	146	8.1	1327	14.1	200	11.4
Non-response	0	0.0	0	0.0	0	0.0	0	0.0	0	0.0
Gender										
Male	7158	55.3	3993	48.0	854	47.3	5437	57.9	867	49.4
Female	5789	44.7	4325	52.0	953	52.7	3948	42.1	888	50.6
Non-response	0	0.0	0	0.0	0	0.0	0	0.0	0	0.0
Race and ethnicity										
Black, non-Hispanic	1097	8.5	1039	12.5	170	9.4	749	8.0	178	10.1
Hispanic	1504	11.6	1561	18.8	237	13.1	1016	10.8	251	14.3
White, non-Hispanic	9493	73.3	5030	60.5	1272	70.4	7014	74.7	1207	68.8
Other, non-Hispanic	499	3.9	370	4.4	77	4.3	346	3.7	76	4.3
2+ races, non-Hispanic	354	2.7	318	3.8	51	2.8	260	2.8	43	2.5
Non-response	0	0.0	0	0.0	0	0.0	0	0.0	0	0.0
Marital status										
Now married	8074	62.4	4460	53.6	1089	60.3	5961	63.5	1024	58.3
Widowed	770	5.9	303	3.6	82	4.5	582	6.2	106	6.0
Divorced	1456	11.2	858	10.3	240	13.3	1010	10.8	206	11.7
Separated	193	1.5	191	2.3	34	1.9	122	1.3	37	2.1
Never married	2454	19.0	2506	30.1	362	20	1710	18.2	382	21.8
Non-response	0	0.0	0	0.0	0	0.0	0	0.0	0	0.0
Education										
No high school diploma or GED	624	4.8	694	8.3	121	6.7	416	4.4	87	5.0
High school graduate or GED	2813	21.7	2084	25.1	452	25	2002	21.3	359	20.5
Some college or Associate degree	3896	30.1	2649	31.8	584	32.3	2773	29.5	539	30.7
Bachelor's degree	3133	24.2	1726	20.8	372	20.6	2337	24.9	424	24.2
Master's degree or higher	2481	19.2	1165	14.0	278	15.4	1857	19.8	346	19.7
Non-response	0	0.0	0	0.0	0	0.0	0	0.0	0	0.0

Table S7, continued.

Characteristic	2022 (Wave1)				Wave1 respondents who left the panel prior to wave2 (n = 1,807)		2023 (Wave2)			
	Respondents (n = 12,947)		Non-respondents (n = 8,318)				Respondents (n = 9,385)		Non-respondents (n = 1,755)	
	Unweighted n	Unweighted %	Unweighted n	Unweighted %	Unweighted n	Unweighted %	Unweighted n	Unweighted %	Unweighted n	Unweighted %
Household Income										
< $10,000	371	2.9	410	4.9	72	4	233	2.5	66	3.8
$10,000 - $24,999	1078	8.3	793	9.5	189	10.5	727	7.7	162	9.2
$25,000 - $49,999	2232	17.2	1558	18.7	318	17.6	1617	17.2	297	16.9
$50,000 - $74,999	2236	17.3	1427	17.2	313	17.3	1631	17.4	292	16.6
$75,000 - $99,999	1999	15.4	1203	14.5	236	13.1	1499	16.0	264	15.0
$100,000 - $149,999	2410	18.6	1461	17.6	336	18.6	1734	18.5	340	19.4
≥ $150,000	2621	20.2	1466	17.6	343	19	1944	20.7	334	19.0
Non-response	0	0.0	0	0.0	0	0.0	0	0.0	0	0.0
Employment										
Working full-time	5645	43.6	4514	54.3	889	49.2	3869	41.2	887	50.5
Working part-time	1620	12.5	1342	16.1	258	14.3	1133	12.1	229	13.0
Not working	5682	43.9	2462	29.6	660	36.5	4383	46.7	639	36.4
Non-response	0	0.0	0	0.0	0	0.0	0	0.0	0	0.0
Census division										
New England	509	3.9	297	3.6	73	4	374	4.0	62	3.5
Mid-Atlantic	1407	10.9	915	11.0	191	10.6	1001	10.7	215	12.3
East-North Central	1878	14.5	1117	13.4	262	14.5	1370	14.6	246	14.0
West-North Central	952	7.4	597	7.2	137	7.6	676	7.2	139	7.9
South Atlantic	2538	19.6	1652	19.9	326	18	1881	20.0	331	18.9
East-South Central	737	5.7	579	7.0	117	6.5	538	5.7	82	4.7
West-South Central	1371	10.6	1093	13.1	207	11.5	965	10.3	199	11.3
Mountain	1125	8.7	573	6.9	156	8.6	825	8.8	144	8.2
Pacific	2430	18.8	1495	18.0	338	18.7	1755	18.7	337	19.2
Non-response	0	0.0	0	0.0	0	0.0	0	0.0	0	0.0

Mean [(SD) ages were as follows: Wave 1 responders, 55.7 (16.7); Wave 1 non-responders, 45.4 (16.8); Wave 1 respondents who left the panel prior to Wave 2, 52.2 (16.2); Wave 2 responders, 57.0 (16.5); Wave 2 non-responders, 52.5 (17.5).

This table first appeared as Table S1 in the supplement to Wintemute GJ, Robinson SL, Crawford A, Tomsich EA, Reeping PM, Shev AB, Velasquez B, Tancredi D. Single-year change in views of democracy and society and support for political violence in the USA: findings from a 2023 nationally representative survey. Injury Epidemiology. 2024;11(1):20.

Table S8. Mean scores and mean change scores across categories of covariates

Characteristic	Unweighted n	Weighted % (95% CI)	2022 score Mean (95% CI)	2023 score Mean (95% CI)	Change score Mean (95% CI)
**Age**					
18-24	310	3.3 (2.9, 3.7)	0.13 (0.01, 0.24)	0.15 (0.01, 0.28)	0.02 (-0.08, 0.13)
25-34	856	9.1 (8.5, 9.7)	0.06 (-0.02, 0.14)	0.12 (0.03, 0.22)	0.06 (-0.02, 0.14)
35-44	1252	13.3 (12.7, 14)	0.00 (-0.06, 0.07)	-0.02 (-0.10, 0.05)	-0.03 (-0.10, 0.05)
45-54	1255	13.4 (12.7, 14.1)	-0.06 (-0.13, 0.00)	-0.13 (-0.19, -0.07)	**-0.07 (-0.13, -0.01)**[†]
55-64	2043	21.8 (20.9, 22.6)	-0.07 (-0.11, -0.04)	-0.09 (-0.13, -0.05)	-0.02 (-0.06, 0.03)
65-74	2342	25 (24.1, 25.8)	-0.02 (-0.06, 0.01)	-0.06 (-0.10, -0.02)	-0.04 (-0.07, 0.00)
75+	1327	14.1 (13.4, 14.8)	0.06 (0.02, 0.11)	0.09 (0.04, 0.14)	0.03 (-0.02, 0.07)
**Gender**					
Female	3866	41.4 (40.4, 42.4)	-0.01 (-0.05, 0.03)	-0.06 (-0.10, -0.02)	**-0.05 (-0.08, -0.01)**
Male	5340	57.2 (56.2, 58.2)	0.00 (-0.04, 0.04)	0.04 (0.00, 0.08)	**0.04 (0.00, 0.07)**
Other	125	1.3 (1.1, 1.6)	0.46 (0.17, 0.76)	0.44 (0.07, 0.81)	-0.03 (-0.25, 0.20)
**Race/Ethnicity**					
White, Non-Hispanic	7014	74.7 (73.9, 75.6)	-0.04 (-0.06, -0.01)	-0.05 (-0.07, -0.02)	-0.01 (-0.04, 0.01)
Black, Non-Hispanic	748	8.0 (7.4, 8.5)	0.16 (0.04, 0.28)	0.20 (0.07, 0.33)	0.04 (-0.06, 0.15)
Hispanic, any race	1016	10.8 (10.2, 11.5)	0.02 (-0.06, 0.09)	-0.03 (-0.11, 0.05)	-0.04 (-0.12, 0.03)
Asian American / Pacific Islander, non-Hispanic	277	3.0 (2.6, 3.3)	-0.01 (-0.15, 0.13)	0.03 (-0.11, 0.18)	0.04 (-0.08, 0.17)
Other race, Non-Hispanic[*]	330	3.5 (3.1, 3.9)	0.17 (-0.01, 0.35)	0.16 (-0.06, 0.38)	-0.01 (-0.20, 0.19)
**Marital status**					
Now married	5961	63.5 (62.5, 64.5)	-0.07 (-0.10, -0.04)	-0.08 (-0.10, -0.05)	-0.01 (-0.03, 0.02)
Widowed	582	6.2 (5.7, 6.7)	0.09 (0.01, 0.17)	-0.03 (-0.12, 0.07)	**-0.12 (-0.20, -0.03)**
Divorced or separated	1132	12.1 (11.4, 12.7)	0.01 (-0.05, 0.07)	0.03 (-0.04, 0.11)	0.02 (-0.05, 0.10)
Never married	1710	18.2 (17.4, 19.0)	0.13 (0.06, 0.19)	0.12 (0.05, 0.19)	-0.01 (-0.07, 0.05)
**Education**					
No high school diploma or GED	416	4.4 (4.0, 4.8)	0.15 (0.03, 0.28)	0.19 (0.05, 0.33)	0.04 (-0.10, 0.17)
High school graduate (high school diploma, or the equivalent GED)	2002	21.3 (20.5, 22.2)	0.14 (0.08, 0.19)	0.08 (0.02, 0.15)	-0.05 (-0.11, 0.00)
Some college or associate degree	2773	29.5 (28.6, 30.5)	-0.01 (-0.05, 0.03)	-0.01 (-0.06, 0.03)	0.00 (-0.04, 0.04)
Bachelor's degree	2337	24.9 (24.0, 25.8)	-0.11 (-0.15, -0.06)	-0.10 (-0.14, -0.05)	0.01 (-0.03, 0.05)
Master’s degree or higher	1857	19.8 (19.0, 20.6)	-0.16 (-0.21, -0.12)	-0.15 (-0.20, -0.10)	0.01 (-0.04, 0.06)

Table S8, continued.

Characteristic	Unweighted n	Weighted % (95% CI)	2022 score Mean (95% CI)	2023 score Mean (95% CI)	Change score Mean (95% CI)
**Household Income**					
Less than $10,000	233	2.5 (2.2, 2.8)	0.48 (0.25, 0.71)	0.51 (0.25, 0.78)	0.03 (-0.22, 0.28)
$10,000 to $24,999	727	7.7 (7.2, 8.3)	0.20 (0.09, 0.30)	0.13 (0.02, 0.24)	-0.07 (-0.17, 0.04)
$25,000 to $49,999	1617	17.2 (16.5, 18)	0.06 (0.00, 0.13)	0.08 (0.02, 0.15)	0.02 (-0.04, 0.08)
$50,000 to $74,999	1631	17.4 (16.6, 18.1)	-0.03 (-0.09, 0.04)	-0.02 (-0.08, 0.05)	0.01 (-0.05, 0.07)
$75,000 to $99,999	1499	16 (15.2, 16.7)	-0.01 (-0.07, 0.06)	-0.03 (-0.10, 0.03)	-0.03 (-0.09, 0.03)
$100,000 to $149,999	1734	18.5 (17.7, 19.3)	-0.06 (-0.11, -0.01)	-0.06 (-0.12, 0.00)	0.00 (-0.06, 0.05)
$150,000 or more	1944	20.7 (19.9, 21.5)	-0.12 (-0.17, -0.08)	-0.14 (-0.20, -0.09)	-0.02 (-0.07, 0.03)
**Employment**					
Working full-time	3869	41.2 (40.2, 42.2)	-0.03 (-0.07, 0.01)	-0.06 (-0.10, -0.02)	-0.03 (-0.06, 0.01)
Working part-time	1133	12.1 (11.4, 12.7)	0.03 (-0.04, 0.10)	0.01 (-0.07, 0.09)	-0.02 (-0.09, 0.05)
Not working, retired	3361	35.8 (34.8, 36.8)	0.00 (-0.03, 0.03)	-0.02 (-0.05, 0.01)	-0.02 (-0.05, 0.01)
Not working, not retired	1022	10.9 (10.3, 11.5)	0.07 (-0.01, 0.15)	0.13 (0.04, 0.23)	0.06 (-0.03, 0.14)
**Census region**					
New England	374	4.0 (3.6, 4.4)	-0.03 (-0.15, 0.08)	-0.06 (-0.19, 0.08)	-0.02 (-0.12, 0.08)
Mid-Atlantic	1001	10.7 (10, 11.3)	-0.06 (-0.12, 0.01)	-0.03 (-0.10, 0.04)	0.03 (-0.04, 0.09)
East-North Central	1370	14.6 (13.9, 15.3)	0.00 (-0.06, 0.07)	-0.03 (-0.10, 0.03)	-0.04 (-0.10, 0.03)
West-North Central	676	7.2 (6.7, 7.7)	0.05 (-0.02, 0.13)	0.04 (-0.05, 0.12)	-0.01 (-0.09, 0.06)
South Atlantic	1881	20 (19.2, 20.9)	0.03 (-0.03, 0.09)	0.01 (-0.06, 0.08)	-0.02 (-0.08, 0.03)
East-South Central	538	5.7 (5.3, 6.2)	0.08 (-0.05, 0.22)	-0.02 (-0.14, 0.11)	-0.10 (-0.25, 0.05)
West-South Central	965	10.3 (9.7, 10.9)	0.02 (-0.07, 0.10)	0.00 (-0.09, 0.09)	-0.02 (-0.10, 0.06)
Mountain	825	8.8 (8.2, 9.4)	-0.03 (-0.09, 0.04)	0.03 (-0.07, 0.12)	0.05 (-0.05, 0.15)
Pacific	1755	18.7 (17.9, 19.5)	-0.01 (-0.08, 0.05)	0.01 (-0.06, 0.09)	0.03 (-0.03, 0.09)
**Children under 17 in household**					
No	7446	79.3 (78.5, 80.2)	-0.01 (-0.03, 0.02)	0.00 (-0.03, 0.03)	0.01 (-0.02, 0.04)
Yes	1939	20.7 (19.8, 21.5)	0.03 (-0.03, 0.09)	-0.02 (-0.08, 0.04)	-0.05 (-0.11, 0.01)

Table S8, continued.

Characteristic	Unweighted n	Weighted % (95% CI)	2022 score Mean (95% CI)	2023 score Mean (95% CI)	Change score Mean (95% CI)
**Political Ideology**					
Extremely liberal	365	4.0 (3.6, 4.4)	0.25 (0.09, 0.42)	0.29 (0.12, 0.46)	0.04 (-0.11, 0.18)
Liberal	1261	13.7 (13, 14.4)	-0.07 (-0.14, -0.01)	-0.16 (-0.22, -0.10)	**-0.09 (-0.15, -0.03)**
Somewhat liberal	828	9.0 (8.4, 9.6)	-0.15 (-0.24, -0.07)	-0.10 (-0.21, 0.01)	0.06 (-0.03, 0.14)
Moderate, middle of the road	2926	31.9 (30.9, 32.8)	-0.06 (-0.11, -0.02)	-0.09 (-0.14, -0.05)	-0.03 (-0.07, 0.02)
Somewhat conservative	1163	12.7 (12.0, 13.4)	0.06 (-0.02, 0.15)	0.14 (0.04, 0.25)	0.08 (0.00, 0.16)
Conservative	2135	23.3 (22.4, 24.1)	0.15 (0.10, 0.20)	0.14 (0.08, 0.20)	-0.01 (-0.06, 0.04)
Extremely conservative	501	5.5 (5.0, 5.9)	0.28 (0.17, 0.40)	0.24 (0.11, 0.37)	-0.04 (-0.17, 0.10)
**Firearm ownership**					
Not a firearm owner	4301	46.2 (45.2, 47.2)	-0.04 (-0.07, 0.00)	-0.05 (-0.09, -0.01)	-0.01 (-0.05, 0.02)
Firearm owner	4381	47.1 (46.1, 48.1)	0.10 (0.07, 0.14)	0.13 (0.09, 0.17)	0.02 (-0.01, 0.06)
Lives in a household with firearms	626	6.7 (6.2, 7.2)	0.03 (-0.04, 0.10)	-0.03 (-0.13, 0.06)	-0.06 (-0.14, 0.02)
**Military service**					
Non-veteran	7649	81.6 (80.8, 82.4)	0.00 (-0.03, 0.02)	-0.01 (-0.04, 0.02)	-0.01 (-0.04, 0.02)
Veteran, non-combat	832	8.9 (8.3, 9.5)	0.06 (-0.01, 0.13)	0.02 (-0.05, 0.10)	-0.04 (-0.11, 0.03)
Combat veteran	893	9.5 (8.9, 10.1)	0.17 (0.07, 0.28)	0.22 (0.10, 0.35)	0.05 (-0.02, 0.13)

Information on the relationship between change scores and change in responses to political violence questions is given in Table S2. For reference, a change score mean of 0.08 (the high score in this table) corresponds to a mean change of -0.01 points per question. A change score mean of -0.09 (the low score in this table) corresponds to a mean change of -0.06 points per question.

[*] American Indian or Alaska Native, Non-Hispanic, 2+ Races, Non-Hispanic, other

[†] Statistically significant change scores ($p < 0.05$) are in bold font

Table S9. Experience of life events and adjusted mean differences in change scores for political violence question subsets

Life event	Justification subset*	Willingness subset*	Firearm subset*
I had a child or a grandchild (yes vs no)	-0.05 (-0.14, 0.04)	-0.12 (-0.24, 0.01)	-0.06 (-0.17, 0.04)
I started a new romantic relationship, or an existing relationship grew stronger (yes vs no)	0.01 (-0.08, 0.10)	0.00 (-0.11, 0.10)	0.03 (-0.08, 0.15)
I got married or engaged (yes vs no)	0.08 (-0.08, 0.23)	0.02 (-0.18, 0.22)	0.12 (-0.07, 0.31)
I got a new job, or a better job (yes vs no)	-0.03 (-0.13, 0.06)	0.02 (-0.11, 0.15)	0.02 (-0.11, 0.15)
I lost my job or had my hours cut back (yes vs no)	0.05 (-0.07, 0.17)	0.17 (0.00, 0.34)	-0.01 (-0.20, 0.19)
I suffered a serious illness (yes vs no)	0.05 (-0.04, 0.15)	-0.02 (-0.14, 0.11)	0.04 (-0.05, 0.14)
My partner, a close family member, or a close friend suffered a serious illness or died (yes vs no)	0.01 (-0.04, 0.07)	0.02 (-0.06, 0.09)	-0.01 (-0.08, 0.06)
Things improved for me financially (yes vs no)	-0.04 (-0.10, 0.02)	**-0.08 (-0.16, 0.00)**†	**-0.07 (-0.14, 0.00)**
Things got worse for me financially (yes vs no)	-0.03 (-0.10, 0.05)	0.00 (-0.10, 0.10)	-0.07 (-0.17, 0.02)
I started a new educational activity or completed one (yes vs no)	0.06 (-0.04, 0.16)	0.10 (-0.03, 0.22)	0.08 (-0.02, 0.19)
I started a new social or community service activity (yes vs no)	0.05 (-0.04, 0.14)	-0.02 (-0.15, 0.11)	0.05 (-0.05, 0.16)
I made some good new friends (yes vs no)	-0.01 (-0.07, 0.05)	0.07 (-0.01, 0.15)	-0.06 (-0.13, 0.02)
I got burned out (yes vs no)	0.00 (-0.06, 0.06)	0.05 (-0.04, 0.14)	-0.08 (-0.17, 0.01)
I was arrested or convicted of a crime, or I spent time in jail or prison (yes vs no)	**0.62 (0.17, 1.07)**	0.41 (-0.31, 1.12)	0.14 (-1.03, 1.32)
I gave up on politics (yes vs no)	0.06 (-0.01, 0.13)	**0.10 (0.02, 0.19)**	0.07 (-0.02, 0.16)
My political beliefs changed a lot (yes vs no)	-0.05 (-0.17, 0.08)	-0.15 (-0.31, 0.00)	-0.14 (-0.32, 0.03)
I decided there was too much violence in my life (yes vs no)	0.07 (-0.10, 0.25)	0.09 (-0.11, 0.29)	-0.11 (-0.31, 0.08)
I had a positive experience with someone I thought was my enemy (yes vs no)	0.09 (-0.03, 0.21)	0.08 (-0.08, 0.23)	0.08 (-0.08, 0.25)

Table S9, continued.

**Life event**	**Justification subset***	**Willingness subset***	**Firearm subset***
**Number of life events**			
0	Reference‡	Reference‡	Reference‡
1	-0.02 (-0.09, 0.06)	0.03 (-0.07, 0.12)	-0.05 (-0.13, 0.03)
2	0.01 (-0.07, 0.08)	**0.11 (0.00, 0.21)**	-0.02 (-0.11, 0.07)
3	-0.05 (-0.13, 0.04)	0.06 (-0.05, 0.18)	-0.07 (-0.18, 0.04)
4	0.06 (-0.04, 0.16)	**0.13 (0.01, 0.26)**	-0.11 (-0.24, 0.01)
≥5	**0.14 (0.04, 0.24)**	**0.25 (0.11, 0.38)**	0.01 (-0.12, 0.14)
**Event cluster§**			
**Cluster 1** (yes vs no)	0.01 (-0.04, 0.07)¶	0.03 (-0.04, 0.10)¶	0.05 (-0.01, 0.12)¶
**Cluster 2** (yes vs no)	0.02 (-0.03, 0.07)	**0.10 (0.03, 0.17)**	**-0.07 (-0.13, 0.00)**
**Cluster 3** (yes vs no)	0.02 (-0.03, 0.07)	0.03 (-0.03, 0.10)	-0.03 (-0.09, 0.04)

Information on the relationship between change scores and change in responses to political violence questions is given in Table S2. This relationship varies with the number of questions included in the calculation and therefore differs across question subsets.

* Results are from a linear regression model which accounts for complex survey weights. Each life event is included in a separate model. Models are adjusted for age, gender, race and ethnicity, education, income, region, marital status, work status, having children in the household, political ideology, firearm ownership, veteran status and all other life events.

† Statistically significant change scores ($P < 0.05$) are in bold font.

‡ Not adjusted for all other life events.

§ Cluster 1 events were both positive and negative and concerned the partner/marriage relationship, family and extended family, employment, and health status. Cluster 2 events were negative and concerned financial status, crime and violence, mental health, and politics. Cluster 3 events were positive and concerned financial status, education, relationships with others, and social networks.

¶ Additionally adjusted for all other clusters.

Table S10. Experience of life events and adjusted mean differences in change scores for political violence question subsets, by gender

	Justification subset*		Willingness subset*		Firearm subset*	
	**Among men** (n = 5340)	**Among women** (n = 3866)	**Among men** (n = 5340)	**Among women** (n = 3866)	**Among men** (n = 5340)	**Among women** (n = 3866)
**Life event**						
I had a child or a grandchild (yes vs no)	-0.07 (-0.19, 0.06)	-0.01 (-0.14, 0.12)	-0.16 (-0.33, 0.01)	-0.07 (-0.24, 0.10)	-0.08 (-0.24, 0.07)	-0.03 (-0.16, 0.10)
I started a new romantic relationship, or an existing relationship grew stronger (yes vs no)	0.00 (-0.10, 0.10)	0.03 (-0.11, 0.16)	0.03 (-0.12, 0.18)	-0.04 (-0.20, 0.11)	0.03 (-0.13, 0.19)	0.06 (-0.10, 0.22)
I got married or engaged (yes vs no)	0.03 (-0.18, 0.24)	0.15 (-0.09, 0.39)	0.06 (-0.22, 0.34)	0.08 (-0.23, 0.38)	0.08 (-0.17, 0.33)	0.21 (-0.05, 0.48)
I got a new job, or a better job (yes vs no)	-0.12 (-0.26, 0.01)	0.03 (-0.11, 0.16)	-0.06 (-0.24, 0.12)	0.07 (-0.11, 0.26)	0.01 (-0.17, 0.18)	-0.02 (-0.20, 0.15)
I lost my job or had my hours cut back (yes vs no)	0.06 (-0.13, 0.24)	0.07 (-0.09, 0.23)	0.16 (-0.08, 0.40)	0.16 (-0.09, 0.41)	-0.11 (-0.42, 0.19)	0.09 (-0.14, 0.32)
I suffered a serious illness (yes vs no)	0.12 (-0.02, 0.25)	-0.02 (-0.16, 0.12)	0.08 (-0.07, 0.23)	-0.09 (-0.28, 0.10)	0.06 (-0.08, 0.20)	0.04 (-0.09, 0.18)
My partner, a close family member, or a close friend suffered a serious illness or died (yes vs no)	-0.03 (-0.12, 0.05)	0.05 (-0.02, 0.13)	-0.06 (-0.18, 0.06)	0.08 (-0.02, 0.18)	-0.08 (-0.18, 0.03)	0.05 (-0.04, 0.13)
Things improved for me financially (yes vs no)	-0.05 (-0.13, 0.03)	-0.05 (-0.14, 0.04)	**-0.12 (-0.23, -0.01)**†	-0.04 (-0.16, 0.07)	**-0.10 (-0.19, 0.00)**	-0.07 (-0.17, 0.03)
Things got worse for me financially (yes vs no)	0.02 (-0.08, 0.12)	-0.06 (-0.16, 0.05)	0.03 (-0.11, 0.16)	-0.01 (-0.15, 0.12)	-0.06 (-0.21, 0.08)	-0.07 (-0.18, 0.04)
I started a new educational activity or completed one (yes vs no)	-0.06 (-0.19, 0.07)	**0.20 (0.05, 0.34)**	0.01 (-0.17, 0.19)	0.17 (-0.01, 0.35)	0.05 (-0.13, 0.22)	0.12 (-0.02, 0.26)
I started a new social or community service activity (yes vs no)	0.14 (-0.03, 0.31)	-0.03 (-0.14, 0.08)	-0.05 (-0.26, 0.16)	0.02 (-0.14, 0.18)	0.12 (-0.08, 0.32)	0.03 (-0.08, 0.14)
I made some good new friends (yes vs no)	0.06 (-0.02, 0.14)	-0.06 (-0.14, 0.02)	**0.15 (0.04, 0.25)**	0.00 (-0.12, 0.11)	0.03 (-0.06, 0.12)	**-0.12 (-0.23, 0.00)**
I got burned out (yes vs no)	0.00 (-0.08, 0.08)	0.00 (-0.09, 0.08)	0.10 (-0.02, 0.22)	0.04 (-0.09, 0.16)	-0.04 (-0.13, 0.06)	-0.11 (-0.25, 0.02)
I was arrested or convicted of a crime, or I spent time in jail or prison (yes vs no)	**0.65 (0.14, 1.16)**	0.46 (-0.45, 1.36)	**0.79 (0.03, 1.55)**	-0.51 (-1.84, 0.82)	0.41 (-1.02, 1.85)	-0.45 (-1.97, 1.08)
I gave up on politics (yes vs no)	0.05 (-0.03, 0.14)	0.06 (-0.04, 0.16)	0.08 (-0.04, 0.21)	0.11 (-0.01, 0.22)	0.04 (-0.11, 0.19)	0.09 (-0.01, 0.18)
My political beliefs changed a lot (yes vs no)	0.03 (-0.12, 0.18)	-0.07 (-0.24, 0.11)	-0.11 (-0.32, 0.09)	-0.13 (-0.35, 0.08)	0.07 (-0.12, 0.26)	**-0.26 (-0.50, -0.02)**
I decided there was too much violence in my life (yes vs no)	0.06 (-0.23, 0.35)	0.08 (-0.14, 0.29)	-0.11 (-0.41, 0.19)	**0.25 (0.01, 0.50)**	**-0.38 (-0.73, -0.04)**	0.07 (-0.13, 0.28)
I had a positive experience with someone I thought was my enemy (yes vs no)	0.00 (-0.16, 0.16)	0.14 (-0.03, 0.31)	-0.15 (-0.37, 0.07)	0.24 (0.03, 0.46)	-0.21 (-0.44, 0.02)	**0.33 (0.10, 0.56)**

Table S10, continued.

	Justification subset		Willingness subset		Firearm subset	
	**Among men** (n = 5340)	**Among women** (n = 3866)	**Among men** (n = 5340)	**Among women** (n = 3866)	**Among men** (n = 5340)	**Among women** (n = 3866)
**Number of life events**						
0	Reference[‡]	Reference[‡]	Reference[‡]	Reference[‡]	Reference[‡]	Reference[‡]
1	**-0.09 (-0.18, 0.00)**	0.06 (-0.05, 0.18)	-0.03 (-0.16, 0.11)	0.09 (-0.05, 0.24)	-0.07 (-0.18, 0.05)	-0.05 (-0.16, 0.07)
2	-0.01 (-0.11, 0.09)	0.03 (-0.08, 0.13)	0.05 (-0.09, 0.20)	**0.16 (0.02, 0.30)**	-0.04 (-0.16, 0.08)	0.00 (-0.12, 0.12)
3	-0.05 (-0.18, 0.07)	-0.05 (-0.16, 0.06)	0.04 (-0.12, 0.20)	0.10 (-0.06, 0.25)	-0.09 (-0.23, 0.05)	-0.04 (-0.20, 0.12)
4	0.05 (-0.08, 0.19)	0.07 (-0.06, 0.21)	0.11 (-0.07, 0.29)	**0.19 (0.00, 0.37)**	-0.05 (-0.22, 0.11)	-0.15 (-0.33, 0.03)
≥5	**0.17 (0.03, 0.32)**	0.13 (-0.01, 0.28)	**0.23 (0.02, 0.43)**	**0.28 (0.10, 0.46)**	0.00 (-0.24, 0.23)	0.03 (-0.12, 0.17)
**Event cluster[§]**						
**Cluster 1** (yes vs no)	-0.03 (-0.09, 0.04)[¶]	0.05 (-0.03, 0.12)[¶]	0.02 (-0.07, 0.12)[¶]	0.03 (-0.07, 0.13)[¶]	-0.03 (-0.11, 0.06)[¶]	0.11 (0.02, 0.20)[¶]
**Cluster 2** (yes vs no)	0.05 (-0.01, 0.12)	-0.01 (-0.08, 0.07)	**0.12 (0.02, 0.21)**	**0.11 (0.01, 0.20)**	-0.02 (-0.10, 0.06)	**-0.10 (-0.19, 0.00)**
**Cluster 3** (yes vs no)	0.03 (-0.04, 0.10)	0.00 (-0.07, 0.08)	-0.01 (-0.11, 0.09)	0.06 (-0.03, 0.16)	-0.05 (-0.14, 0.05)	-0.02 (-0.11, 0.07)

Information on the relationship between change scores and change in responses to political violence questions is given in Table S2. This relationship varies with the number of questions included in the calculation and therefore differs across question subsets.

[*] Results are from a linear regression model which accounts for complex survey weights. Each life event is included in a separate model. Models are adjusted for age, gender, race and ethnicity, education, income, region, marital status, work status, having children in the household, political ideology, firearm ownership, veteran status and all other life events.

[†] Statistically significant change scores ($P < 0.05$) are in bold font.

[‡] Not adjusted for all other life events.

[§] Cluster 1 events were both positive and negative and concerned the partner/marriage relationship, family and extended family, employment, and health status. Cluster 2 events were negative and concerned financial status, crime and violence, mental health, and politics. Cluster 3 events were positive and concerned financial status, education, relationships with others, and social networks.

[¶] Additionally adjusted for all other clusters.

Table S11. Experience of life events and adjusted mean differences in change scores for political violence question subsets, among respondents who indicated violence was usually or always justified to advance at least 1 political objective in 2022

Life event	Justification subset*	Willingness subset*	Firearm subset*
I had a child or a grandchild (yes vs no)	0.02 (-0.17, 0.21)	-0.11 (-0.29, 0.06)	-0.15 (-0.38, 0.08)
I started a new romantic relationship, or an existing relationship grew stronger (yes vs no)	0.00 (-0.18, 0.18)	0.13 (-0.03, 0.30)	-0.01 (-0.29, 0.28)
I got married or engaged (yes vs no)	-0.02 (-0.34, 0.31)	-0.04 (-0.34, 0.26)	0.14 (-0.23, 0.51)
I got a new job, or a better job (yes vs no)	-0.10 (-0.32, 0.12)	-0.20 (-0.44, 0.04)	-0.01 (-0.33, 0.31)
I lost my job or had my hours cut back (yes vs no)	0.02 (-0.21, 0.26)	0.05 (-0.16, 0.27)	-0.03 (-0.40, 0.33)
I suffered a serious illness (yes vs no)	0.13 (-0.07, 0.33)	-0.10 (-0.27, 0.07)	0.01 (-0.17, 0.19)
My partner, a close family member, or a close friend suffered a serious illness or died (yes vs no)	0.02 (-0.10, 0.14)	0.06 (-0.06, 0.18)	0.01 (-0.14, 0.16)
Things improved for me financially (yes vs no)	-0.10 (-0.22, 0.03)	-0.02 (-0.15, 0.11)	-0.10 (-0.26, 0.05)
Things got worse for me financially (yes vs no)	-0.01 (-0.16, 0.13)	0.04 (-0.10, 0.18)	**-0.22 (-0.42, -0.01)** †
I started a new educational activity or completed one (yes vs no)	0.10 (-0.13, 0.34)	0.14 (-0.07, 0.35)	0.16 (-0.08, 0.40)
I started a new social or community service activity (yes vs no)	0.01 (-0.21, 0.24)	-0.17 (-0.37, 0.04)	0.09 (-0.18, 0.36)
I made some good new friends (yes vs no)	-0.03 (-0.16, 0.10)	-0.07 (-0.20, 0.06)	-0.07 (-0.26, 0.11)
I got burned out (yes vs no)	0.02 (-0.12, 0.15)	0.06 (-0.08, 0.21)	-0.11 (-0.30, 0.07)
I was arrested or convicted of a crime, or I spent time in jail or prison (yes vs no)	**0.78 (0.12, 1.45)**	0.39 (-0.63, 1.41)	0.03 (-1.81, 1.86)
I gave up on politics (yes vs no)	0.02 (-0.11, 0.15)	0.09 (-0.04, 0.21)	0.11 (-0.07, 0.28)
My political beliefs changed a lot (yes vs no)	0.02 (-0.21, 0.24)	-0.13 (-0.35, 0.10)	-0.25 (-0.57, 0.06)
I decided there was too much violence in my life (yes vs no)	0.06 (-0.25, 0.36)	-0.11 (-0.39, 0.18)	-0.32 (-0.69, 0.05)
I had a positive experience with someone I thought was my enemy (yes vs no)	0.23 (-0.02, 0.48)	0.18 (-0.05, 0.41)	0.15 (-0.23, 0.52)
**Number of life events**			
0	Reference‡	Reference‡	Reference‡
1	-0.08 (-0.24, 0.08)	0.01 (-0.14, 0.16)	-0.10 (-0.32, 0.12)
2	0.01 (-0.16, 0.18)	**0.17 (0.01, 0.34)**	-0.04 (-0.28, 0.20)
3	-0.11 (-0.31, 0.09)	-0.07 (-0.26, 0.12)	-0.05 (-0.33, 0.23)
4	0.03 (-0.18, 0.23)	-0.01 (-0.23, 0.20)	-0.25 (-0.56, 0.06)
≥5	**0.23 (0.01, 0.45)**	**0.23 (0.01, 0.44)**	-0.07 (-0.37, 0.23)
**Event cluster**§			
**Cluster 1** (yes vs no)	0.09 (-0.02, 0.20)¶	0.00 (-0.11, 0.11)¶	0.08 (-0.06, 0.23)¶
**Cluster 2** (yes vs no)	0.07 (-0.04, 0.18)	**0.13 (0.02, 0.24)**	-0.14 (-0.29, 0.01)
**Cluster 3** (yes vs no)	0.00 (-0.12, 0.11)	-0.01 (-0.11, 0.10)	-0.03 (-0.17, 0.11)

Information on the relationship between change scores and change in responses to political violence questions is given in Table S2. This relationship varies with the number of questions included in the calculation and therefore differs across question subsets.

[*] Results are from a linear regression model which accounts for complex survey weights. Each life event is included in a separate model. Models are adjusted for age, gender, race and ethnicity, education, income, region, marital status, work status, having children in the household, political ideology, firearm ownership, veteran status and all other life events.

[†] Statistically significant change scores ($P < 0.05$) are in bold font.

[‡] Not adjusted for all other life events.

[§] Cluster 1 events were both positive and negative and concerned the partner/marriage relationship, family and extended family, employment, and health status. Cluster 2 events were negative and concerned financial status, crime and violence, mental health, and politics. Cluster 3 events were positive and concerned financial status, education, relationships with others, and social networks.

[¶] Additionally adjusted for all other clusters.

Table S12. Experience of life events and adjusted mean differences in change scores for political violence question subsets, among respondents who strongly or very strongly approved of at least one extremist organization/movement in 2022

Life event	Justification subset*	Willingness subset*	Firearm subset*
I had a child or a grandchild (yes vs no)	0.13 (-0.25, 0.50)	-0.13 (-0.53, 0.28)	**-0.45 (-0.90, 0.00)**†
I started a new romantic relationship, or an existing relationship grew stronger (yes vs no)	-0.10 (-0.41, 0.22)	0.08 (-0.27, 0.42)	0.35 (-0.04, 0.74)
I got married or engaged (yes vs no)	0.24 (-0.25, 0.72)	0.04 (-0.48, 0.56)	0.27 (-0.23, 0.77)
I got a new job, or a better job (yes vs no)	-0.18 (-0.48, 0.13)	-0.19 (-0.54, 0.17)	-0.02 (-0.43, 0.39)
I lost my job or had my hours cut back (yes vs no)	0.06 (-0.32, 0.43)	0.15 (-0.27, 0.58)	-0.08 (-0.65, 0.49)
I suffered a serious illness (yes vs no)	0.17 (-0.18, 0.52)	-0.04 (-0.36, 0.27)	0.12 (-0.22, 0.46)
My partner, a close family member, or a close friend suffered a serious illness or died (yes vs no)	-0.03 (-0.25, 0.20)	-0.11 (-0.37, 0.15)	-0.11 (-0.40, 0.17)
Things improved for me financially (yes vs no)	0.02 (-0.22, 0.26)	-0.03 (-0.30, 0.25)	-0.04 (-0.32, 0.23)
Things got worse for me financially (yes vs no)	-0.16 (-0.43, 0.12)	-0.08 (-0.39, 0.22)	**-0.39 (-0.75, -0.03)**
I started a new educational activity or completed one (yes vs no)	-0.01 (-0.32, 0.30)	0.28 (-0.10, 0.66)	-0.07 (-0.36, 0.21)
I started a new social or community service activity (yes vs no)	0.00 (-0.27, 0.28)	-0.21 (-0.52, 0.11)	-0.06 (-0.34, 0.22)
I made some good new friends (yes vs no)	-0.05 (-0.29, 0.18)	-0.10 (-0.38, 0.17)	0.21 (-0.10, 0.52)
I got burned out (yes vs no)	0.13 (-0.06, 0.32)	0.09 (-0.15, 0.33)	-0.15 (-0.43, 0.13)
I was arrested or convicted of a crime, or I spent time in jail or prison (yes vs no)	0.71 (-0.22, 1.64)	-0.09 (-1.44, 1.27)	-0.40 (-2.61, 1.81)
I gave up on politics (yes vs no)	0.14 (-0.12, 0.40)	**0.34 (0.02, 0.65)**	0.18 (-0.20, 0.56)
My political beliefs changed a lot (yes vs no)	-0.35 (-0.74, 0.03)	**-0.63 (-1.12, -0.13)**	-0.46 (-1.10, 0.18)
I decided there was too much violence in my life (yes vs no)	0.07 (-0.42, 0.57)	-0.12 (-0.71, 0.46)	-0.60 (-1.22, 0.01)
I had a positive experience with someone I thought was my enemy (yes vs no)	0.08 (-0.24, 0.40)	0.07 (-0.32, 0.45)	-0.21 (-0.66, 0.24)
**Number of life events**			
0	Reference‡	Reference‡	Reference‡
1	0.12 (-0.27, 0.51)	-0.08 (-0.49, 0.33)	-0.01 (-0.44, 0.42)
2	0.02 (-0.32, 0.37)	-0.16 (-0.57, 0.25)	0.22 (-0.18, 0.62)
3	0.13 (-0.26, 0.52)	-0.17 (-0.64, 0.29)	0.05 (-0.34, 0.45)
4	-0.19 (-0.59, 0.22)	**-0.55 (-1.04, -0.06)**	-0.36 (-0.85, 0.14)
≥5	0.30 (-0.09, 0.69)	-0.01 (-0.49, 0.46)	-0.09 (-0.55, 0.37)
**Event cluster**§			
Cluster 1 (yes vs no)	0.14 (-0.07, 0.35)¶	-0.03 (-0.27, 0.21)¶	0.18 (-0.09, 0.46)¶
Cluster 2 (yes vs no)	0.00 (-0.22, 0.22)	0.07 (-0.17, 0.31)	**-0.39 (-0.67, -0.12)**
Cluster 3 (yes vs no)	0.04 (-0.18, 0.25)	-0.13 (-0.38, 0.12)	0.08 (-0.18, 0.34)

Information on the relationship between change scores and change in responses to political violence questions is given in Table S2. This relationship varies with the number of questions included in the calculation and therefore differs across question subsets.

[*] Results are from a linear regression model which accounts for complex survey weights. Each life event is included in a separate model. Models are adjusted for age, gender, race and ethnicity, education, income, region, marital status, work status, having children in the household, political ideology, firearm ownership, veteran status and all other life events.

[†] Statistically significant change scores ($P < 0.05$) are in bold font.

[‡] Not adjusted for all other life events.

[§] Cluster 1 events were both positive and negative and concerned the partner/marriage relationship, family and extended family, employment, and health status. Cluster 2 events were negative and concerned financial status, crime and violence, mental health, and politics. Cluster 3 events were positive and concerned financial status, education, relationships with others, and social networks.

[¶] Additionally adjusted for all other clusters.

Figure S1. Scree plots for the first factor of the PCA in 2023 for all 35 political violence measures combined and for the justification, willingness, and firearm subsets

a. All measures combined

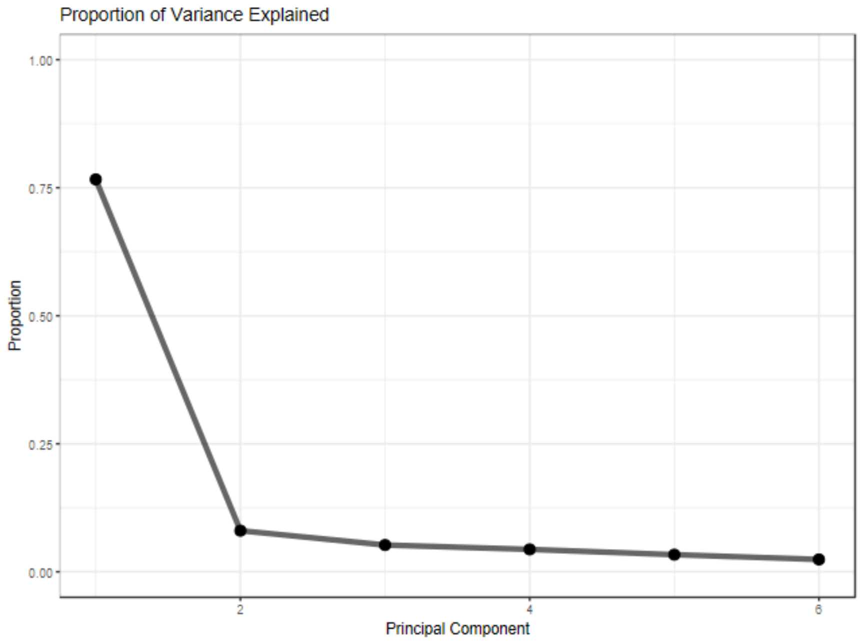

b. Justification subset

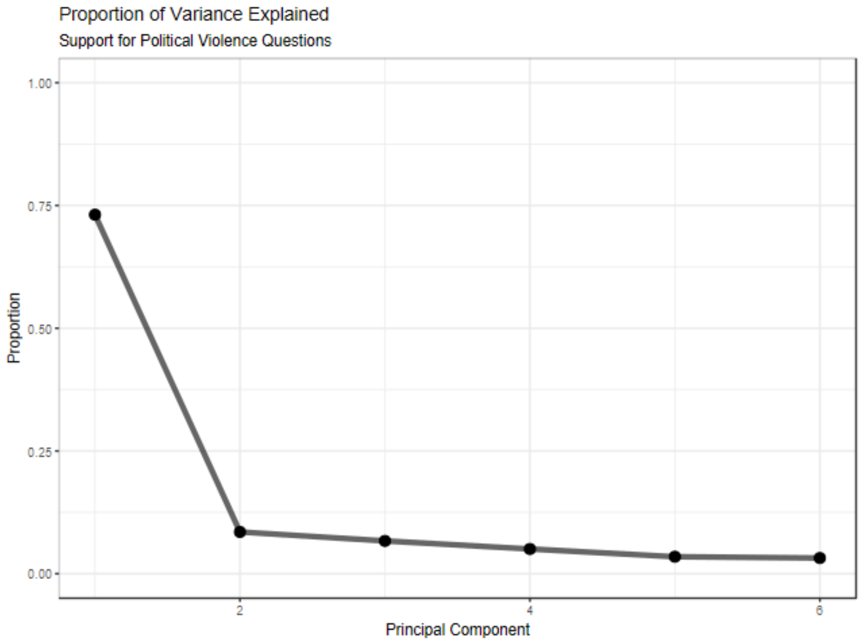

c. Willingness subset

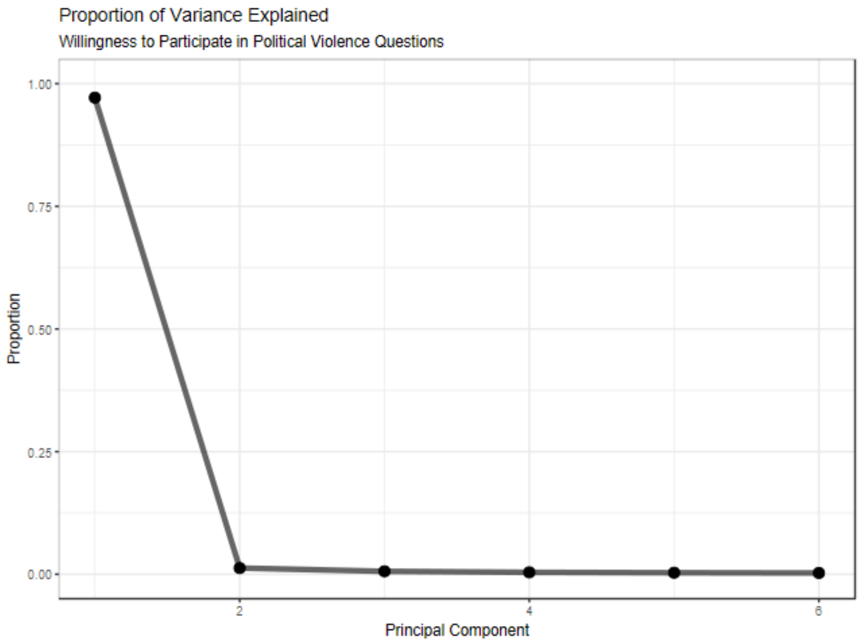

d. Firearm subset

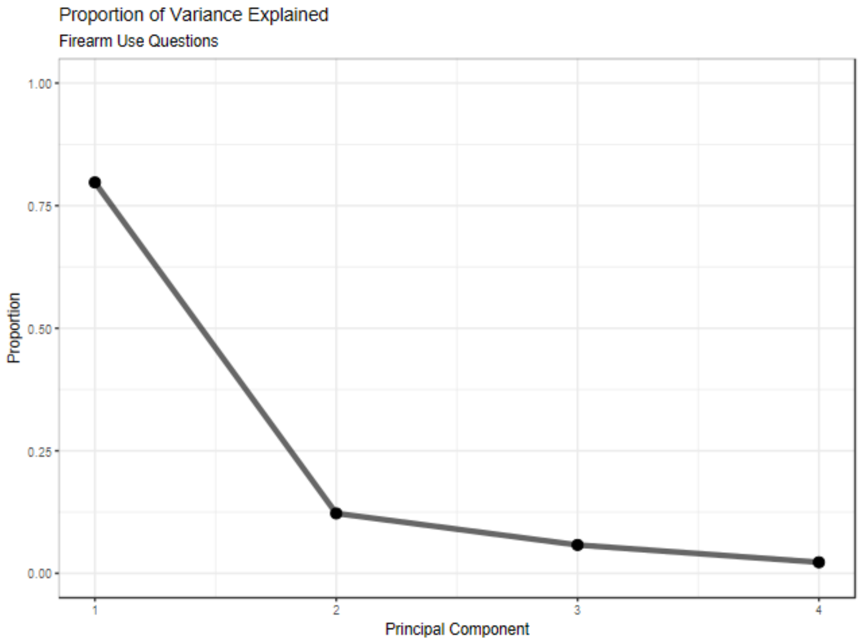

Figure S2. Distribution of change scores for the main analysis

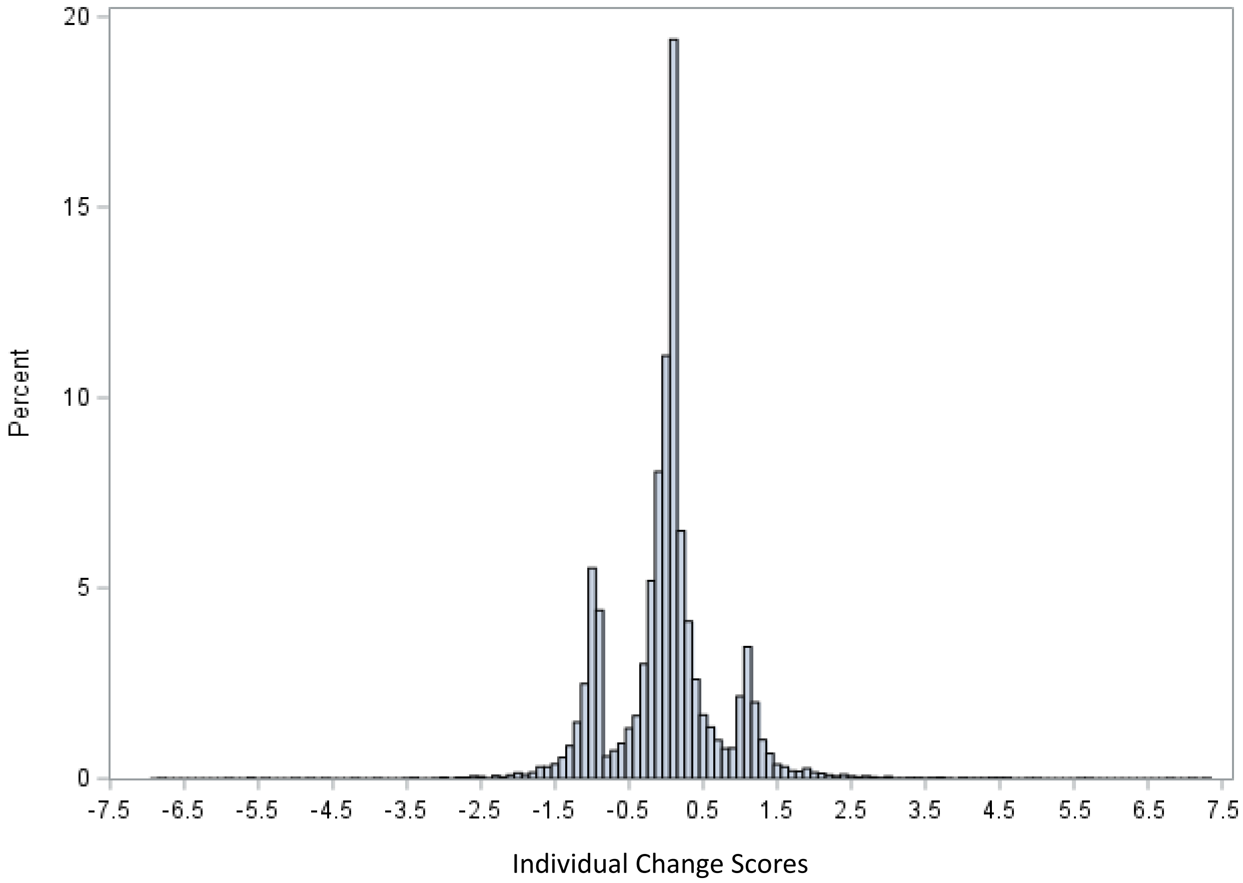

Figure S3. Cluster summaries* and inter-cluster correlations for all cluster solutions

Cluster Summary for 1 Cluster

Cluster	Members	Cluster Variation	Variation Explained	Proportion Explained	Second Eigenvalue
1	18	18	2.719032	0.1511	1.5278

Total variation explained = 2.719032 Proportion = 0.1511

Cluster Summary for 2 Clusters

Cluster	Members	Cluster Variation	Variation Explained	Proportion Explained	Second Eigenvalue
1	11	11	2.296922	0.2088	1.3615
2	7	7	1.792103	0.256	1.0577

Total variation explained = 4.089025 Proportion = 0.2272

Inter-Cluster Correlations

Cluster	1	2
1	1	0.29523
2	0.29523	1

Cluster Summary for 3 Clusters

Cluster	Members	Cluster Variation	Variation Explained	Proportion Explained	Second Eigenvalue
1	7	7	2.095704	0.2994	1.0056
2	6	6	1.698814	0.2831	1.0182
3	5	5	1.61905	0.3238	0.939

Total variation explained = 5.413567 Proportion = 0.3008

Inter-Cluster Correlations

Cluster	1	2	3
1	1	0.25786	0.23638
2	0.25786	1	0.18198
3	0.23638	0.18198	1

Cluster Summary for 4 Clusters

Cluster	Members	Cluster Variation	Variation Explained	Proportion Explained	Second Eigenvalue
1	7	7	2.095704	0.2994	1.0056
2	4	4	1.467345	0.3668	0.995
3	4	4	1.505733	0.3764	0.9384
4	3	3	1.352309	0.4508	0.8727

Total variation explained = 6.421091 Proportion = 0.3567

Inter-Cluster Correlations

Cluster	1	2	3	4
1	1	0.19874	0.22008	0.24254
2	0.19874	1	0.08402	0.26427
3	0.22008	0.08402	1	0.21557
4	0.24254	0.26427	0.21557	1

Cluster Summary for 5 Clusters

Cluster	Members	Cluster Variation	Variation Explained	Proportion Explained	Second Eigenvalue
1	4	4	1.747412	0.4369	0.8474
2	4	4	1.467345	0.3668	0.995
3	4	4	1.505733	0.3764	0.9384
4	3	3	1.352309	0.4508	0.8727
5	3	3	1.298086	0.4327	0.8794

Total variation explained = 7.370886 Proportion = 0.4095

Inter-Cluster Correlations

Cluster	1	2	3	4	5
1	1	0.18358	0.23358	0.22641	0.34429
2	0.18358	1	0.08402	0.26427	0.13987
3	0.23358	0.08402	1	0.21557	0.106
4	0.22641	0.26427	0.21557	1	0.16124
5	0.34429	0.13987	0.106	0.16124	1

* Each summary explains the variation in each group of variables, the number of variables in each cluster, and the proportion of variance explained by each cluster component, with larger values indicating a better fit for the cluster variables.
